# Supplementary material for: Early warning signal for a tipping point suggested by a millennial Atlantic Multidecadal Variability reconstruction
Source: Nat Commun. 2022 Sep 2;13:5176. doi: 10.1038/s41467-022-32704-3 (PMC9440003; doi:10.1038/s41467-022-32704-3)
Supplement: Supplementary file 1 — Supplementary Information [file 41467_2022_32704_MOESM1_ESM.docx]

Supplementary Table 1. **Proxy records added to the PAGES 2k database^36^ to build the PAGES2k+ (P2k+) database (References in Supplementary Note 2).**

| **Model name** | **Research center (Country)** |
| --- | --- |
| BNU-ESM | BNU (China) |
| CCSM4 | NCAR (USA) |
| CESM1-BGC | NCAR (USA) |
| CESM1-CAM5 | NCAR (USA) |
| CNRM-CM5 | CNRM (France) |
| CSIRO-MK3-6-0 | CSIRO (Australia) |
| CanESM2 | CCCma (Canada) |
| EC-Earth | ECMWF (Europe) |
| FGOALS-s2 | CAS (China) |
| FGOALS-g2 | CAS (China) |
| FIO-ESM | QLNM (China) |
| GFDL-CM3 | GFDL (UK) |
| GFL-ESM2G | GFDL (UK) |
| GFDL-ESM2M | CCCma (Canada) |
| GISS-E2-H-CC | NASA GISS (USA) |
| GISS-E2-H | NASA GISS (USA) |
| GISS-E2-R-CC | NASA GISS (USA) |
| GISS-E2-R | NASA GISS (USA) |
| HadGEM2-AO | MOHC (UK) |
| HadGEM2-CC | MOHC (UK) |
| HadGEM2-ES | MOHC (UK) |
| IPSL-CM5A-LR | ISPL (France) |
| IPSL-CM5A-MR | ISPL (France) |
| IPSL-CM5B-LR | ISPL (France) |
| MIROC-ESM-CHEM | JAMSTEC (Japan) |
| MIROC-ESM | JAMSTEC (Japan) |
| MIROC5 | JAMSTEC (Japan) |
| MPI-ESM-LR | MPI (Germany) |
| MPI-ESM-MR | MPI (Germany) |
| MRI-GCM3 | MRI (Japan) |
| NorESM1-ME | NCC (Norway) |
| NorESM1-M | NCC (Norway) |

Supplementary Table 2. **List of the 32 models from the Coupled Model Intercomparison Project phase 5 (CMIP5) used for the estimation of the North Atlantic Sea Surface Temperature (NASST) forced component (Supplementary Fig. 1, Methods).**

| **Model name** | **Research center (Country)** |
| --- | --- |
| ACCESS-CM2 | ACCESS (Australia) |
| ACCESS-ESM1-5 | ACCESS (Australia) |
| BCC-CM2-MR | BCC (China) |
| BCC-ESM1 | BCC (China) |
| CAMS-CSM1-0 | CAMS (China) |
| CESM2 | NCAR (USA) |
| CESM2-FV2 | NCAR (USA) |
| CESM2-WACCM | NCAR (USA) |
| CIESM | THU (China) |
| CMCC-CM2-HR4 | CMCC (Italy) |
| CMCC-CM2-SR5 | CMCC (Italy) |
| CMCC-ESM2 | CMCC (Italy) |
| CanESM5 | CCCma (Canada) |
| FGOALS-f3-L | CAS (China) |
| FGOALS-g3 | CAS (China) |
| FIO-ESM-2-0 | QLNM (China) |
| GFDL-CM4 | GFDL (UK) |
| GFDL-ESM4 | GFDL (UK) |
| GISS-E2-1-H | NASA GISS (USA) |
| IPSL-CM6A-LR | IPSL (France) |
| MCM-UA-1-0 | UA (USA) |
| MIROC6 | JAMSTEC (Japan) |
| MPI-ESM1-2-HAM | MPI (Germany) |
| MPI-ESM1-2-HR | MPI (Germany) |
| MPI-ESM1-2-LR | MPI (Germany) |
| MRI-ESM2-0 | MRI (Japan) |
| NESM3 | NUIST (China) |
| NorCPM1 | NCC (Norway) |
| NorESM2-LM | NCC (Norway) |
| NorESM2-MM | NCC (Norway) |

Supplementary Table 3. **List of the 30 models from the Coupled Model Intercomparison Project phase 6 (CMIP6) used for the estimation of the North Atlantic Sea Surface Temperature (NASST) forced component for the sensitivity study on the use of CMIP5 or CMIP6 (Supplementary Fig. 2-5).**


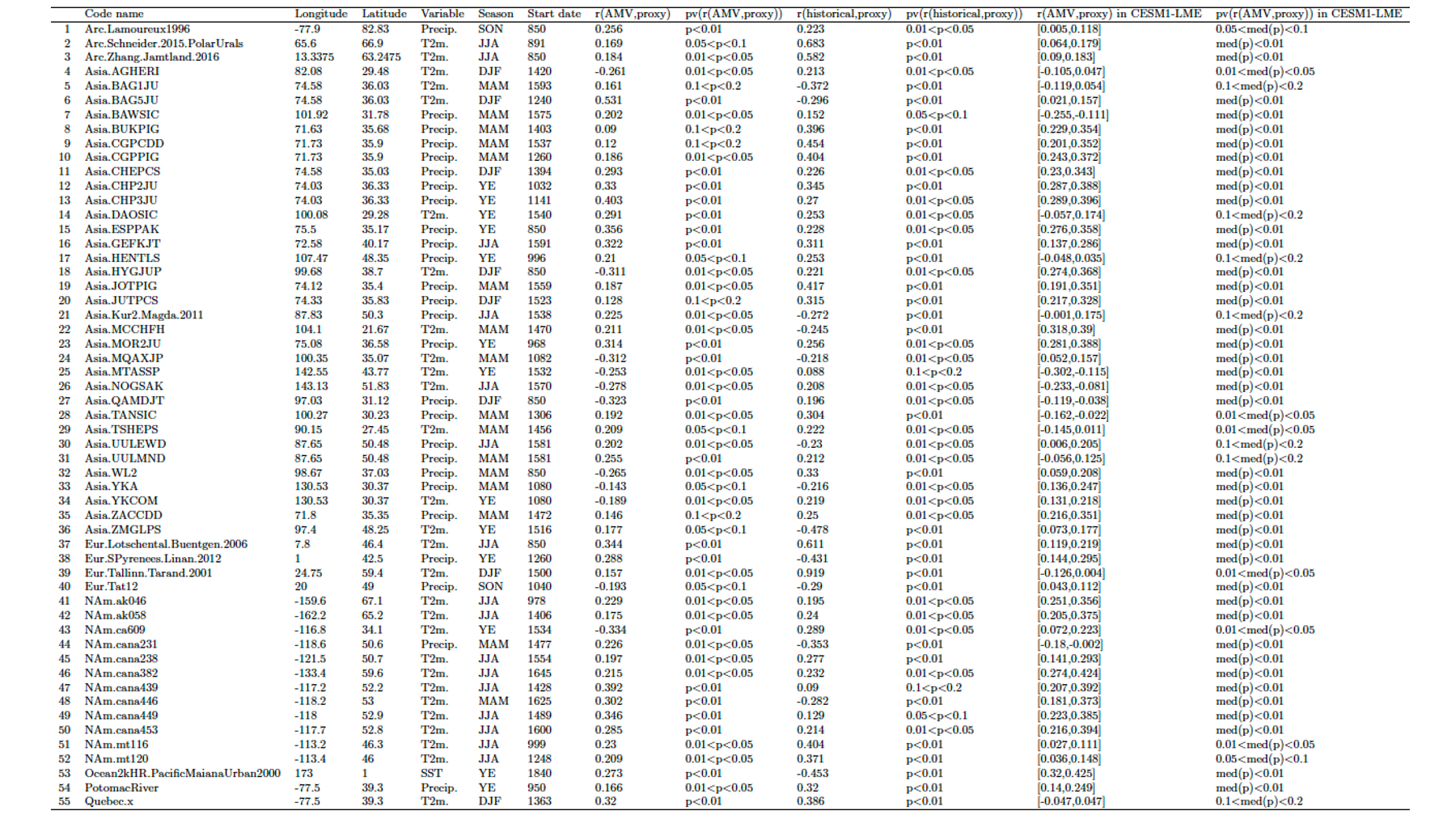


Supplementary Table 4. **Characteristics of proxy records used for the final reconstruction presented in this study.** The correlation metric is denoted $r$, and the p-value of the correlation metric is denoted $\mathrm{pv}(r)$. Historical correlations and their significance have been calculated using the CRUTS4 dataset^37^ for the period 1901-2020. Correlations and their significance for the Community Earth System Model Last Millennium Ensemble (CESM-LME) experiment have been calculated for the period 850-1990, for the 12 members used in this study.

| **Volcano** | **Location** | **Year of the eruption** |
| --- | --- | --- |
| **Unknown** | **Unknown** | **1108** |
| **Unknown** | **Unknown** | **1171** |
| **Unknown** | **Unknown** | **1230** |
| **Samalas** | **Indonesia** | **1257** |
| **Kuwae** | **Vanuatu** | **1453** |
| **Huaynaputina** | **Peru** | **1601** |
| **Parker** | **Philippines** | **1641** |
| **Lakagigar** | **Iceland** | **1783** |
| **Tambora** | **Indonesia** | **1815** |

Supplementary Table 5. **10 last millennium largest volcanic eruptions^38^.**

| **Rank** | **AMV Index** | **Regression method** | **Oldest year for calibration** | **Mean** $\boldsymbol{S}_{\mathbf{CE}}$ **score (3 digits rounded)** |
| --- | --- | --- | --- | --- |
| **1** | $\mathbf{AM}\mathbf{V}_{\mathbf{F}}$ | **RF** | **1987** | **0.226** |
| **2** | $\mathbf{AM}\mathbf{V}_{\mathbf{T}}$ | **PLS** | **1987** | **0.213** |
| **3** | $\mathbf{AM}\mathbf{V}_{\mathbf{F}}$ | **ENET** | **1992** | **0.212** |
| **4** | $\mathbf{AM}\mathbf{V}_{\mathbf{F}}$ | **ENET** | **1990** | **0.208** |
| **5** | $\mathbf{AM}\mathbf{V}_{\mathbf{F}}$ | **ENET** | **1991** | **0.201** |
| **6** | $\mathbf{AM}\mathbf{V}_{\mathbf{F}}$ | **PLS** | **1987** | **0.199** |
| **7** | $\mathbf{AM}\mathbf{V}_{\mathbf{F}}$ | **ENET** | **1979** | **0.199** |
| **8** | $\mathbf{AM}\mathbf{V}_{\mathbf{T}}$ | **ENET** | **1990** | **0.198** |
| **9** | $\mathbf{AM}\mathbf{V}_{\mathbf{F}}$ | **ENET** | **1987** | **0.197** |
| **10** | $\mathbf{AM}\mathbf{V}_{\mathbf{T}}$ | **RF** | **1975** | **0.194** |
| **11** | $\mathbf{AM}\mathbf{V}_{\mathbf{T}}$ | **ENET** | **1987** | **0.194** |
| **12** | $\mathbf{AM}\mathbf{V}_{\mathbf{F}}$ | **ENET** | **1988** | **0.193** |
| **13** | $\mathbf{AM}\mathbf{V}_{\mathbf{F}}$ | **RF** | **1988** | **0.191** |
| **14** | $\mathbf{AM}\mathbf{V}_{\mathbf{T}}$ | **PCR** | **1987** | **0.188** |
| **15** | $\mathbf{AM}\mathbf{V}_{\mathbf{T}}$ | **ENET** | **1988** | **0.185** |
| **16** | $\mathbf{AM}\mathbf{V}_{\mathbf{F}}$ | **ENET** | **1985** | **0.184** |
| **17** | $\mathbf{AM}\mathbf{V}_{\mathbf{F}}$ | **ENET** | **1989** | **0.184** |
| **18** | $\mathbf{AM}\mathbf{V}_{\mathbf{F}}$ | **PCR** | **1985** | **0.183** |
| **19** | $\mathbf{AM}\mathbf{V}_{\mathbf{F}}$ | **ENET** | **1978** | **0.182** |
| **20** | $\mathbf{AM}\mathbf{V}_{\mathbf{F}}$ | **RF** | **1985** | **0.182** |
| **21** | $\mathbf{AM}\mathbf{V}_{\mathbf{T}}$ | **PCR** | **1990** | **0.180** |
| **22** | $\mathbf{AM}\mathbf{V}_{\mathbf{F}}$ | **PCR** | **1992** | **0.180** |
| **23** | $\mathbf{AM}\mathbf{V}_{\mathbf{TS}}$ | **ENET** | **1978** | **0.179** |
| **24** | $\mathbf{AM}\mathbf{V}_{\mathbf{TS}}$ | **ENET** | **1990** | **0.177** |
| **25** | $\mathbf{AM}\mathbf{V}_{\mathbf{F}}$ | **PLS** | **1988** | **0.177** |
| **26** | $\mathbf{AM}\mathbf{V}_{\mathbf{F}}$ | **PCR** | **1988** | **0.176** |
| **27** | $\mathbf{AM}\mathbf{V}_{\mathbf{T}}$ | **ENET** | **1992** | **0.174** |
| **28** | $\mathbf{AM}\mathbf{V}_{\mathbf{TS}}$ | **ENET** | **1982** | **0.173** |
| **29** | $\mathbf{AM}\mathbf{V}_{\mathbf{T}}$ | **PCR** | **1978** | **0.173** |
| **30** | $\mathbf{AM}\mathbf{V}_{\mathbf{F}}$ | **PCR** | **1987** | **0.173** |

Supplementary Table 6. **30 best Atlantic Multidecadal Variability (AMV) reconstruction from this study.** Statistical methods are Principal Component Regression (PCR), Partial Least Squares (PLS), Elastic-Net (ENET), and Random Forest (RF).

| **Rank** | **AMV Index** | **Regression method** | **Oldest year for calibration** | **EWS** $\tau$**statistics (WL=200)** | **EWS** $\tau$**statistics (WL=300)** | **EWS** $\tau$**statistics (WL=400)** |
| --- | --- | --- | --- | --- | --- | --- |
| **1** | $\mathbf{AM}\mathbf{V}_{\mathbf{F}}$ | **RF** | **1987** | **0.62**  **(p<0.01)** | **0.64**  **(p<0.01)** | **0.67**  **(p<0.01)** |
| **2** | $\mathbf{AM}\mathbf{V}_{\mathbf{T}}$ | **PLS** | **1987** | **0.6**  **(p<0.01)** | **0.6**  **(p<0.01)** | **0.58**  **(p<0.01)** |
| **3** | $\mathbf{AM}\mathbf{V}_{\mathbf{F}}$ | **ENET** | **1992** | **0.55**  **(p<0.01)** | **0.52**  **(p<0.01)** | **0.53**  **(p<0.01)** |
| **4** | $\mathbf{AM}\mathbf{V}_{\mathbf{F}}$ | **ENET** | **1990** | **0.65**  **(p<0.01)** | **0.71**  **(p<0.01)** | **0.72**  **(p<0.01)** |
| **5** | $\mathbf{AM}\mathbf{V}_{\mathbf{F}}$ | **ENET** | **1991** | **0.55**  **(p<0.01)** | **0.57**  **(p<0.01)** | **0.59**  **(p<0.01)** |
| **6** | $\mathbf{AM}\mathbf{V}_{\mathbf{F}}$ | **PLS** | **1987** | **0.63**  **(p<0.01)** | **0.66**  **(p<0.01)** | **0.67**  **(p<0.01)** |
| **7** | $\mathbf{AM}\mathbf{V}_{\mathbf{F}}$ | **ENET** | **1979** | **0.73**  **(p<0.01)** | **0.75**  **(p<0.01)** | **0.77**  **(p<0.01)** |
| **8** | $\mathbf{AM}\mathbf{V}_{\mathbf{T}}$ | **ENET** | **1990** | **0.57**  **(p<0.01)** | **0.56**  **(p<0.01)** | **0.54**  **(p<0.01)** |
| **9** | $\mathbf{AM}\mathbf{V}_{\mathbf{F}}$ | **ENET** | **1987** | **0.63**  **(p<0.01)** | **0.65**  **(p<0.01)** | **0.63**  **(p<0.01)** |
| **10** | $\mathbf{AM}\mathbf{V}_{\mathbf{T}}$ | **RF** | **1975** | **0.64**  **(p<0.01)** | **0.66**  **(p<0.01)** | **0.66**  **(p<0.01)** |
| **11** | $\mathbf{AM}\mathbf{V}_{\mathbf{T}}$ | **ENET** | **1987** | **0.61**  **(p<0.01)** | **0.63**  **(p<0.01)** | **0.64**  **(p<0.01)** |
| **12** | $\mathbf{AM}\mathbf{V}_{\mathbf{F}}$ | **ENET** | **1988** | **0.69**  **(p<0.01)** | **0.73**  **(p<0.01)** | **0.75**  **(p<0.01)** |
| **13** | $\mathbf{AM}\mathbf{V}_{\mathbf{F}}$ | **RF** | **1988** | **0.53**  **(p<0.01)** | **0.55**  **(p<0.01)** | **0.55**  **(p<0.01)** |
| **14** | $\mathbf{AM}\mathbf{V}_{\mathbf{T}}$ | **PCR** | **1987** | **0.57**  **(p<0.01)** | **0.56**  **(p<0.01)** | **0.51 (0.1<p<0.2)** |
| **15** | $\mathbf{AM}\mathbf{V}_{\mathbf{T}}$ | **ENET** | **1988** | **0.59**  **(p<0.01)** | **0.6**  **(p<0.01)** | **0.57**  **(p<0.01)** |
| **16** | $\mathbf{AM}\mathbf{V}_{\mathbf{F}}$ | **ENET** | **1985** | **0.65**  **(p<0.01)** | **0.69**  **(p<0.01)** | **0.69**  **(p<0.01)** |
| **17** | $\mathbf{AM}\mathbf{V}_{\mathbf{F}}$ | **ENET** | **1989** | **0.69**  **(p<0.01)** | **0.73**  **(p<0.01)** | **0.74**  **(p<0.01)** |
| **18** | $\mathbf{AM}\mathbf{V}_{\mathbf{F}}$ | **PCR** | **1985** | **0.58**  **(p<0.01)** | **0.56 (p<0.01)** | **0.56**  **(p<0.01)** |
| **19** | $\mathbf{AM}\mathbf{V}_{\mathbf{F}}$ | **ENET** | **1978** | **0.72**  **(p<0.01)** | **0.75**  **(p<0.01)** | **0.76**  **(p<0.01)** |
| **20** | $\mathbf{AM}\mathbf{V}_{\mathbf{F}}$ | **RF** | **1985** | **0.46**  **(p<0.01)** | **0.45**  **(p<0.01)** | **0.43**  **(p<0.01)** |
| **21** | $\mathbf{AM}\mathbf{V}_{\mathbf{T}}$ | **PCR** | **1990** | **0.53**  **(p<0.01)** | **0.49**  **(p<0.01)** | **0.41**  **(p<0.01)** |
| **22** | $\mathbf{AM}\mathbf{V}_{\mathbf{F}}$ | **PCR** | **1992** | **0.46**  **(p<0.01)** | **0.42**  **(p<0.01)** | **0.39**  **(p<0.01)** |
| **23** | $\mathbf{AM}\mathbf{V}_{\mathbf{TS}}$ | **ENET** | **1978** | **0.61**  **(p<0.01)** | **0.66**  **(p<0.01)** | **0.69**  **(p<0.01)** |
| **24** | $\mathbf{AM}\mathbf{V}_{\mathbf{TS}}$ | **ENET** | **1990** | **0.68**  **(p<0.01)** | **0.72**  **(p<0.01)** | **0.75**  **(p<0.01)** |
| **25** | $\mathbf{AM}\mathbf{V}_{\mathbf{F}}$ | **PLS** | **1988** | **0.67**  **(p<0.01)** | **0.68**  **(p<0.01)** | **0.70**  **(p<0.01)** |
| **26** | $\mathbf{AM}\mathbf{V}_{\mathbf{F}}$ | **PCR** | **1988** | **0.59**  **(p<0.01)** | **0.63**  **(p<0.01)** | **0.63**  **(p<0.01)** |
| **27** | $\mathbf{AM}\mathbf{V}_{\mathbf{T}}$ | **ENET** | **1992** | **0.55**  **(p<0.01)** | **0.56**  **(p<0.01)** | **0.56**  **(p<0.01)** |
| **28** | $\mathbf{AM}\mathbf{V}_{\mathbf{TS}}$ | **ENET** | **1982** | **0.68**  **(p<0.01)** | **0.73**  **(p<0.01)** | **0.76**  **(p<0.01)** |
| **29** | $\mathbf{AM}\mathbf{V}_{\mathbf{T}}$ | **PCR** | **1978** | **0.57**  **(p<0.01)** | **0.58**  **(p<0.01)** | **0.55**  **(p<0.01)** |
| **30** | $\mathbf{AM}\mathbf{V}_{\mathbf{F}}$ | **PCR** | **1987** | **0.62 (p<0.01)** | **0.59 (p<0.01)** | **0.57 (p<0.01)** |

Supplementary Table 7. **Kendall statistics for the Early warning signal tests^39,40^ of the 30 best Atlantic Multidecadal Variability (AMV) reconstructions.** Statistical methods are Principal Component Regression (PCR), Partial Least Squares (PLS), Elastic-Net (ENET), and Random Forest (RF).

| **R package** | **Reference** |
| --- | --- |
| biwavelet | 41 |
| glmnet | 42 |
| gplots | 43 |
| latex2exp | 44 |
| maps | 45 |
| ncdf4 | 46 |
| pls | 47 |
| pracma | 48 |
| randomForest | 49 |
| RColorBrewer | 50 |
| sp | 51,52 |
| stringr | 53 |
| VGAM | 54,55 |
| zoo | 56 |

Supplementary Table 8. **List of R packages used for the study.**


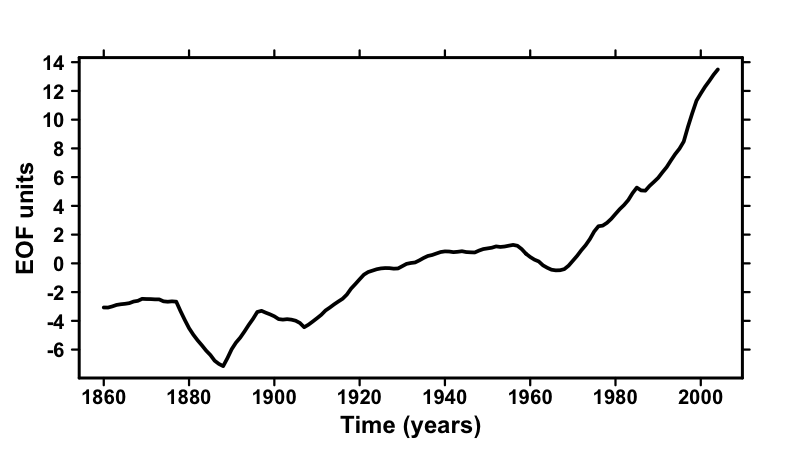


Supplementary Fig. 1. **Estimated forced component of historical North Atlantic Sea Surface Temperature (NASST).** Estimation is performed using signal-to-noise maximizing Empirical Orthogonal Function (EOF)^57^ (*cf*. Methods), using historical simulations from 30 climate models from the Coupled Model Intercomparison Project phase 5 (CMIP5) (Supplementary Table 2). The time series is filtered with 10-year kernel smoothing.

**
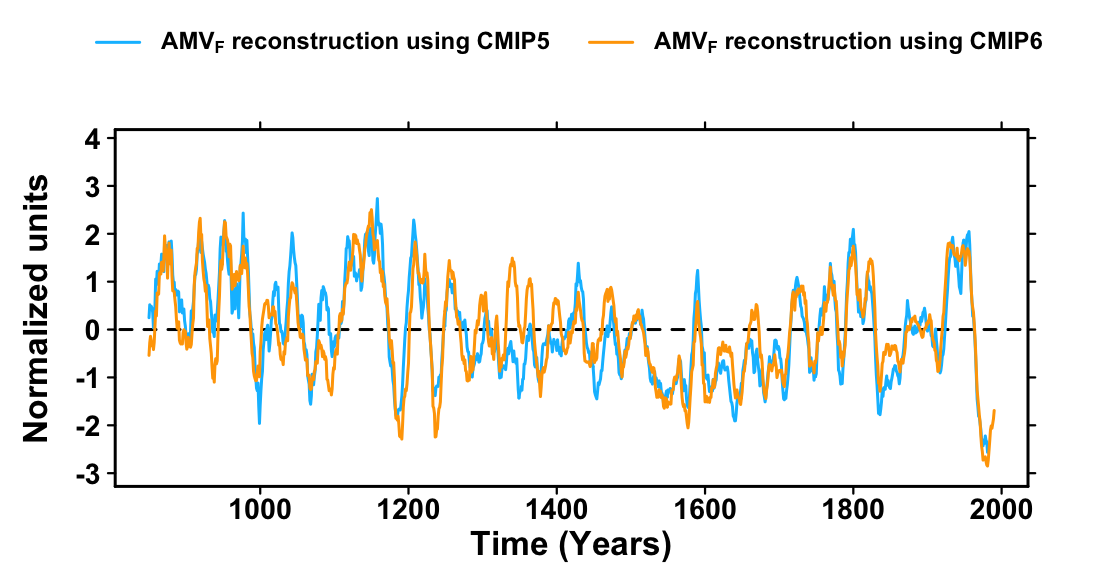
**

Supplementary Fig. 2. **Sensitivity analysis of the Coupled Model Intercomparison Project (CMIP)** **generation used to remove the external forcing.** Best reconstruction obtained with CMIP5 (blue) and CMIP6 (orange) Atmosphere-Ocean General Circulation Model (AOGCM) generations to remove the external forcing from proxy records.


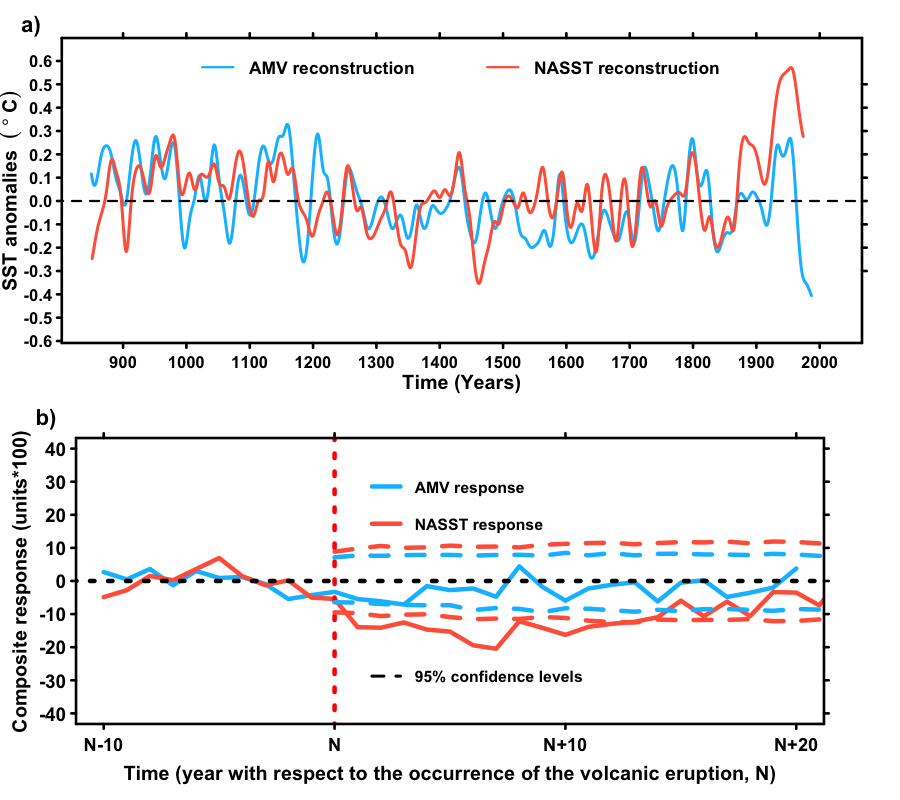


Supplementary Fig. 3. **Same as Fig. 3 but with Coupled Model Intercomparison Project** **phase 6** **(CMIP6)** **generation used to remove external forcing from proxy records to produce the reconstruction (Supplementary Fig. 2). a** Final reconstructions of Atlantic Multidecadal Variability (AMV, blue) and North Atlantic Sea Surface Temperatures (NASST, red), in Sea Surface Temperature anomalies (°C). **b** Superposed epoch analysis^45^ for responses of the AMV and NASST reconstructions to the ten largest eruptions^58^ of the last millennium (see Supplementary Table 5). Composite series are performed for 31 years, with the 11^th^ year being the year of the eruptions. Each individual response is centered to its values 10 years before the eruption (from N-10 to N-1, where N describes the year of occurrence of the eruption) before computing the composite time series. 95% confidence levels have been calculated using a Monte-Carlo approach^45^.


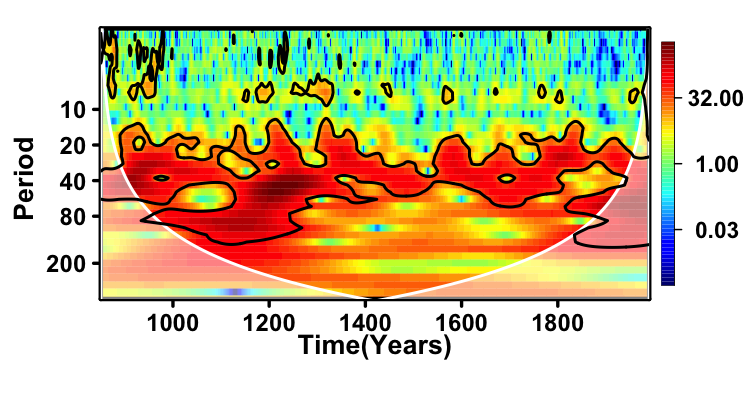


Supplementary Fig. 4. **Same as Fig. 4 but with Coupled Model Intercomparison Project phase 6 (CMIP6) generation used to remove external forcing from proxy records to produce the reconstruction (Supplementary Fig. 2).** Contours provide the 90% confidence level of significance. The white line and the light white-shaded area below indicate the cone of influence. The cone of influence gives the spectrum borders where the edge effect (*ie.* the time boundary effect) becomes too important, which cannot be robustly interpreted.


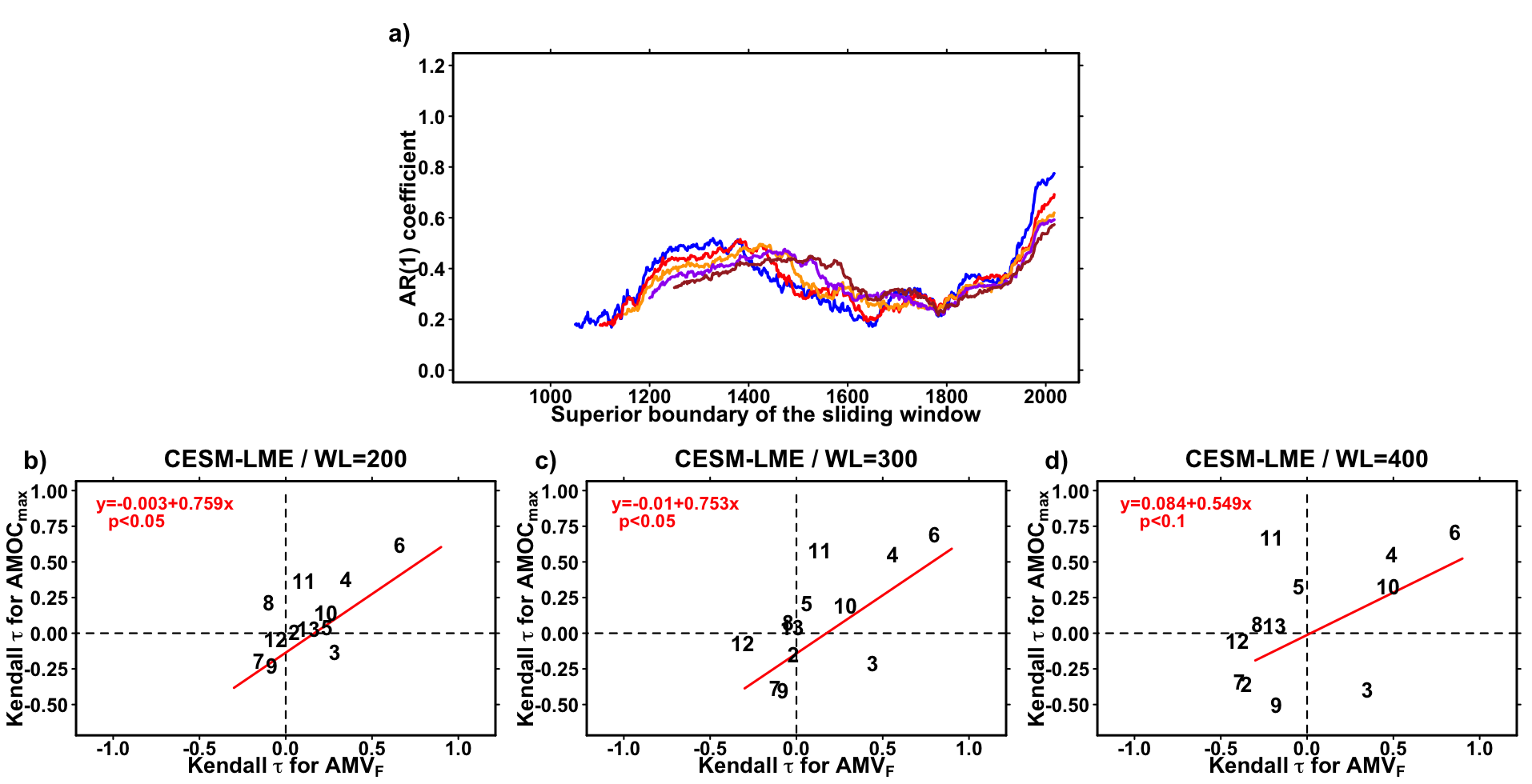


Supplementary Fig. 5. **Same as Fig. 6 but with Coupled Model Intercomparison Project phase 6** **(CMIP6)** **generation used to remove external forcing from proxy records to produce the reconstruction (Supplementary Fig. 2).** Early Warning Signal (EWS) test of the Atlantic Multidecadal Variability reconstructed index (AMV_F_) and relevance in Community Earth System Model Last Millennium Ensemble (CESM-LME) simulations. **a** Early Warning Signal (EWS) for the reconstruction. The applied test is based on first order autoregressive coefficients (AR(1)), for different window lengths (WL)^38,39^. For each WL, sliding AR(1) coefficient are computed and a Kendall τ statistics between time and the sliding AR(1) time series are calculated (see Methods). Significances are approximated using Gaussian distributions because of the large length (>50) of the AR(1) coefficients (see Methods).  **b-d** EWS statistics in Community Earth System Model Last Millennium Ensemble (CESM-LME) simulations. Kendall τ statistics obtained for the maximum Atlantic Meridional Overturning Circulation strength below 500 meters depth (AMOC_max_) AR(1) coefficients (ordinates) are plotted against the Kendall τ statistics obtained for the Atlantic Multidecadal Variability reconstructed index (AMV_F_) AR(1) coefficients (abscissas) for WL=200 (b), WL=300 (c), and WL=400 (d), respectively. Plotted numbers indicate the member index of the CESM-LME simulations (from 2 to 13). Red lines are the ordinary least squares regression lines, and their significance is calculated using a two-tailed Student *t*-test of the regression slope.

**
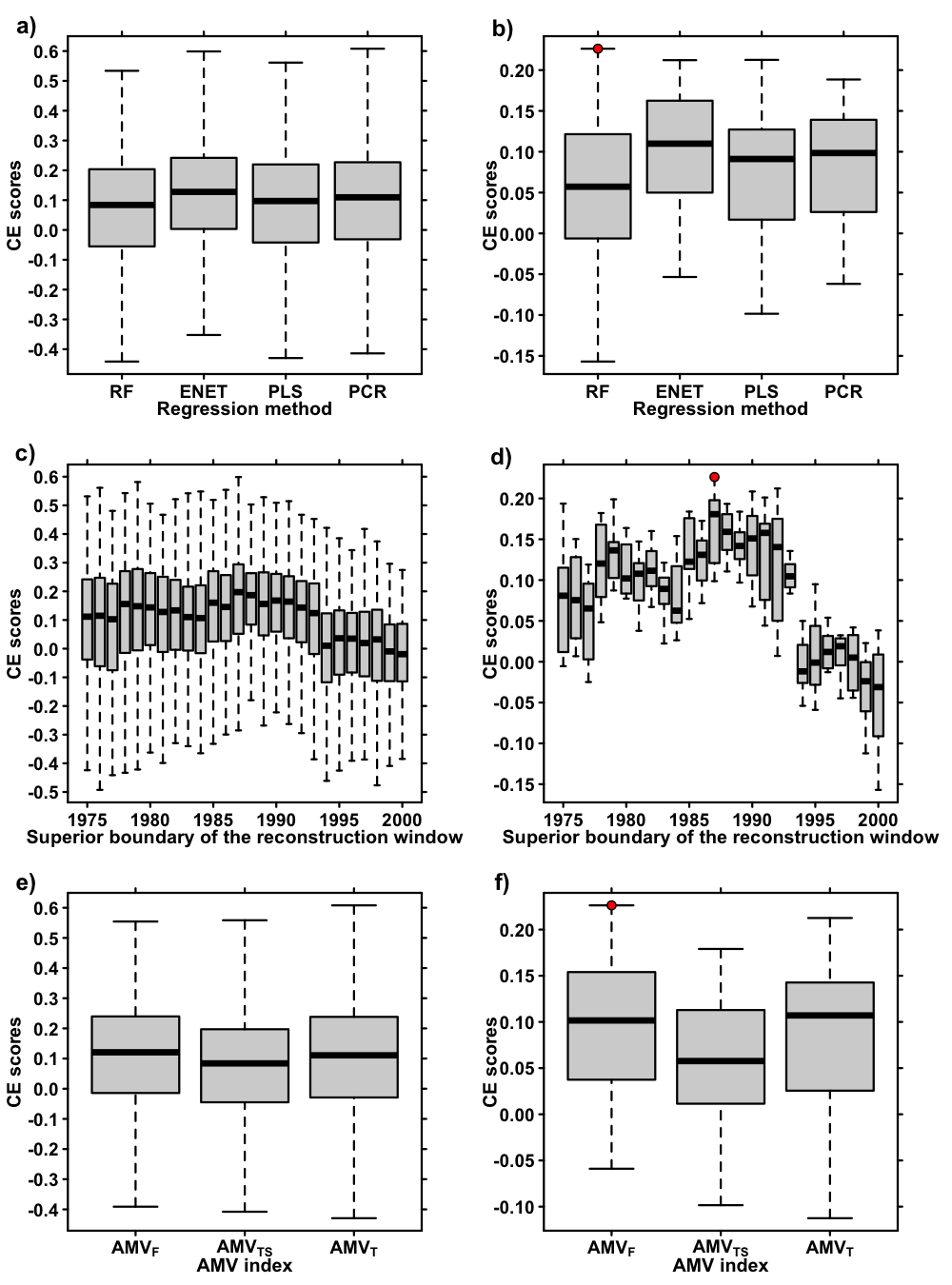
**

Supplementary Fig. 6. **Score by level of inputs for the 312 reconstructions.** Coefficient of Efficiency^58,59^ scores ($S_{\mathrm{CE}}$ or CE here) for the 312 reconstructions compared in this study for the different sources of methodological choices: regression method, reconstruction frame and Atlantic Multidecadal Variability (AMV) index. **a** and **b** give the $S_{\mathrm{CE}}$ scores by regression methods (312/4=78 reconstructions by method). **c** and **d** give the $S_{\mathrm{CE}}$ scores by superior boundary of the reconstruction window (312/26=12 final reconstructions by window). **e** and **f** give the $S_{\mathrm{CE}}$ scores by AMV index (312/3=104 final reconstructions by index). **a**, **c** and **e** give the $S_{\mathrm{CE}}$ scores for all the training splits (312*30=9,360 scores, respectively 2340, 360, and 3120 scores by boxplot for the three panels). **b**, **d** and **f** give $S_{\mathrm{CE}}$ scores for each final reconstruction as the averages of the 30 corresponding individual $S_{\mathrm{CE}}$ scores (312 average scores). Red dots indicate the highest average score obtained for the particular level of input. For all boxplots, medians are shown as heavy dark lines. Boxplots edges give first and third quartiles. Boxplot “whiskers” give the 10%-90% range. Outliers are not shown. A point from a boxplot is here considered as an outlier when it is outside 1.5 times the interquartile range above the upper quartile and below the lower quartile.

**
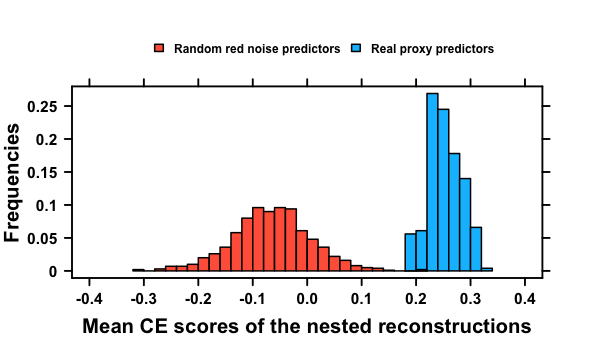
**

Supplementary Fig. 7. **Comparison of** $\boldsymbol{S}_{\mathbf{CE}}$ **(or CE here) scores with those obtained from red noise predictors.** Blue bars are the mean $S_{\mathrm{CE}}$ scores obtained using the real proxy records with the best reconstruction (Supplementary Fig. 6) over the different timeframes of the nested reconstruction (Methods). Red bars are the mean $S_{\mathrm{CE}}$ scores for each timeframe of the nested reconstruction but with simulated red noise instead of the proxy records^60^ (Methods).

**
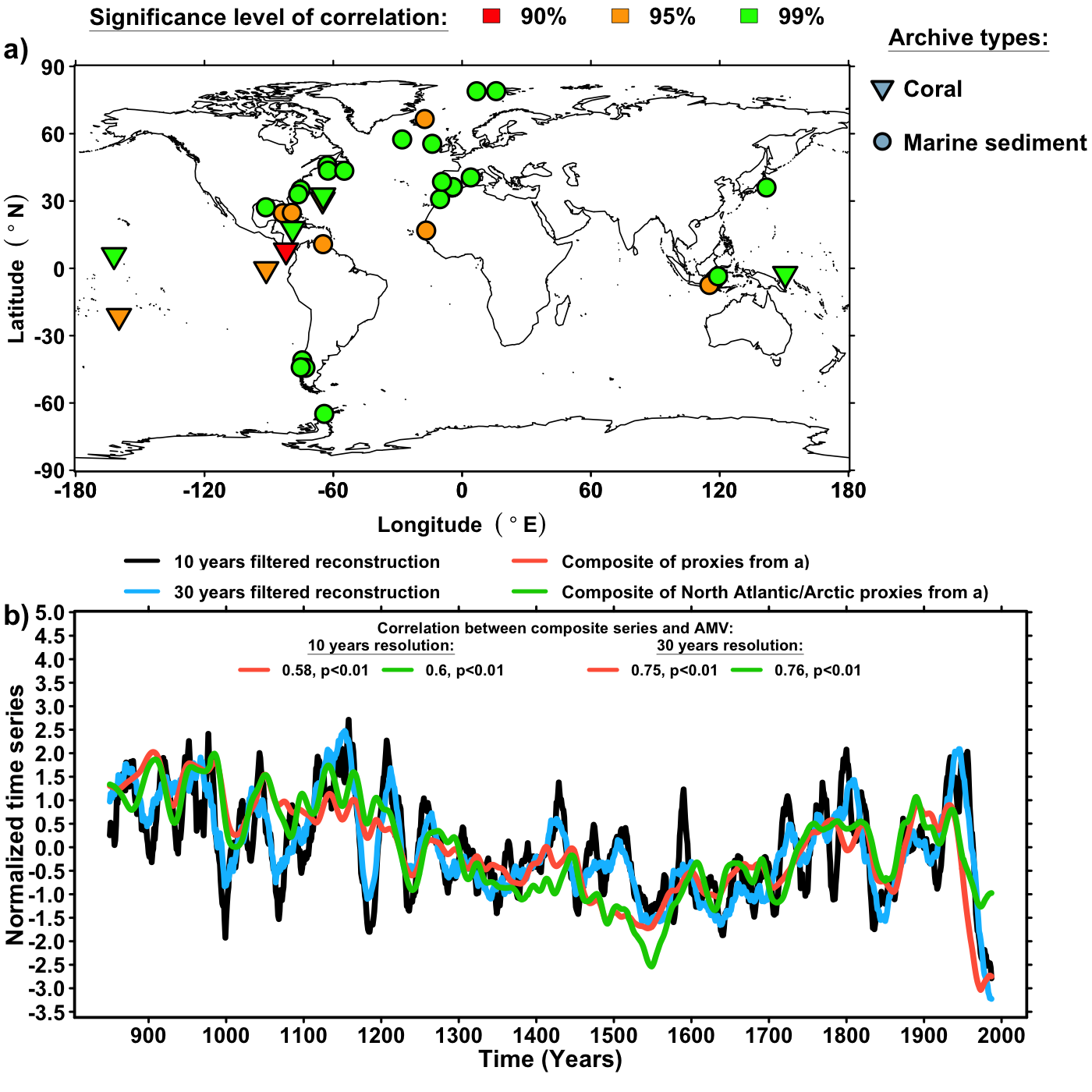
**

Supplementary Fig. 8. **Comparison with independent ocean proxy records. a** 37 Ocean 2k proxy records^36^ significantly correlated at least at the 90% confidence with the AMV over the pre-industrial period (*i.e.* prior to 1870). Coral and marine sediment proxies are indicated as reversed triangle and circle symbols, respectively. **b** 10-years (black) and 30-years (blue) kernel smooth of the Atlantic Multidecadal Variability reconstruction. The red line indicates the composite of all proxy records from panel **a**, and the green line indicate the composite of proxies from panel **a** that only belong to the North Atlantic (including Mediterranean Sea).


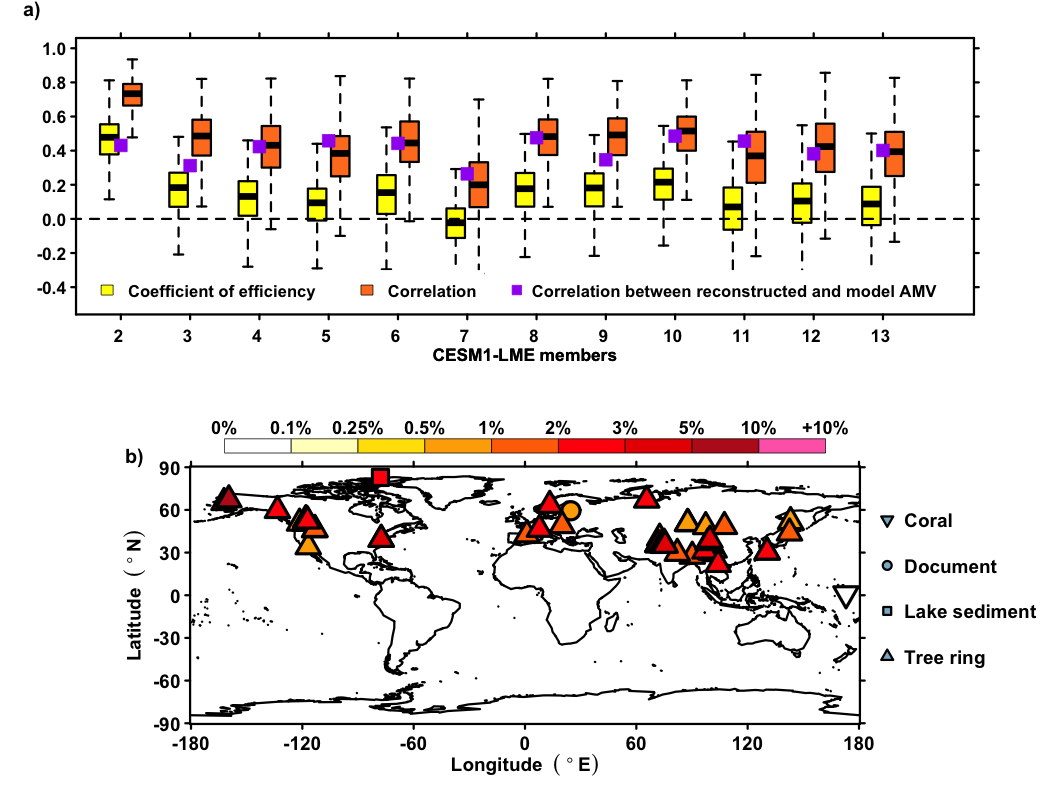


Supplementary Fig. 9. **Calibration-constrained Pseud-Proxy Experiment (PPE) validation.** **a** Coefficient of efficiency scores ($S_{\mathrm{CE}}$) (yellow boxplots), correlation scores (orange boxplots) and correlation between the model Atlantic Multidecadal Variability (AMV) and the reconstructed AMV within the model simulations (Methods) (purple line) for 12 members of Community Earth System Model Last Millennium Ensemble (CESM-LME). **b** Ensemble average of weights of the proxy records from the model simulations from 12 members of CESM-LME (Methods). Coral, document, lake sediment, and tree ring proxies are indicated as reversed triangle, circle, square and triangle symbols, respectively.


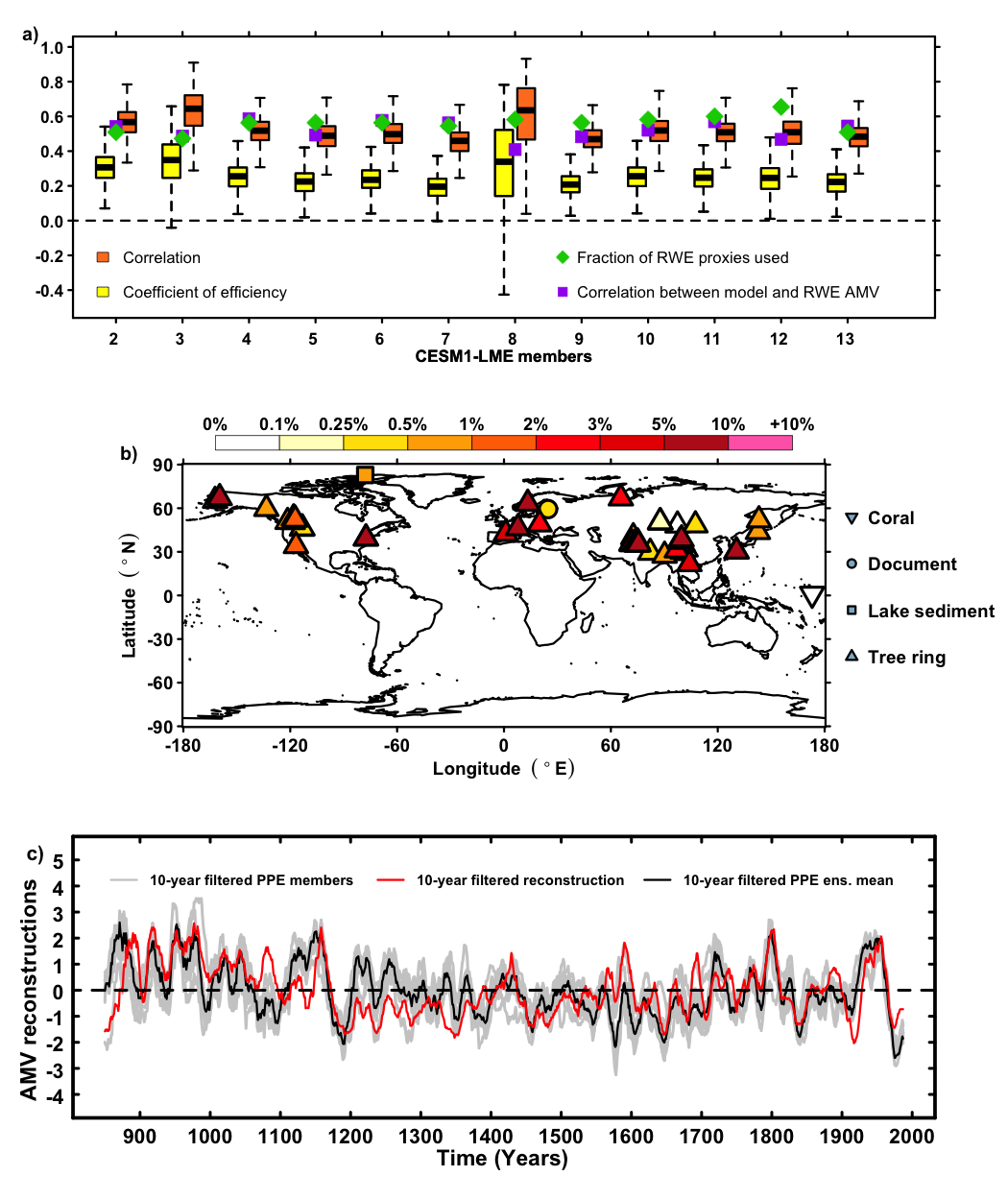


Supplementary Fig. 10. **Model-constrained Pseud-Proxy Experiment (PPE) validation. a** Coefficient of efficiency scores ($S_{\mathrm{CE}}$) scores (yellow boxplots), correlation scores (orange boxplots) and correlation between the real-world experiment (RWE) Atlantic Multidecadal Variability index ($\mathrm{AM}V_{F}$) reconstruction and model-constrained $\mathrm{AM}V_{F}$ reconstructions (Methods) (purple line) for the 12 members of the Community Earth System Model Last Millennium Ensemble (CESM-LME). Green line indicates the fraction of proxy records from the real experiments used in the PPE (see Methods). **b** ensemble average of weights of the proxy records from the real-world experiment for RF trained within the model simulations (Methods). **c** Grey lines: 10-years kernel smooth of the 12 model-based experiment, based on each CESM1-LME member. Black: 10-years kernel smooth of the ensemble average of the 12 model-based reconstructions. Red: 10-years kernel smooth of the reconstruction from the real experiment. Coral, document, lake sediment, and tree ring proxies are indicated as reversed triangle, circle, square and triangle symbols, respectively.


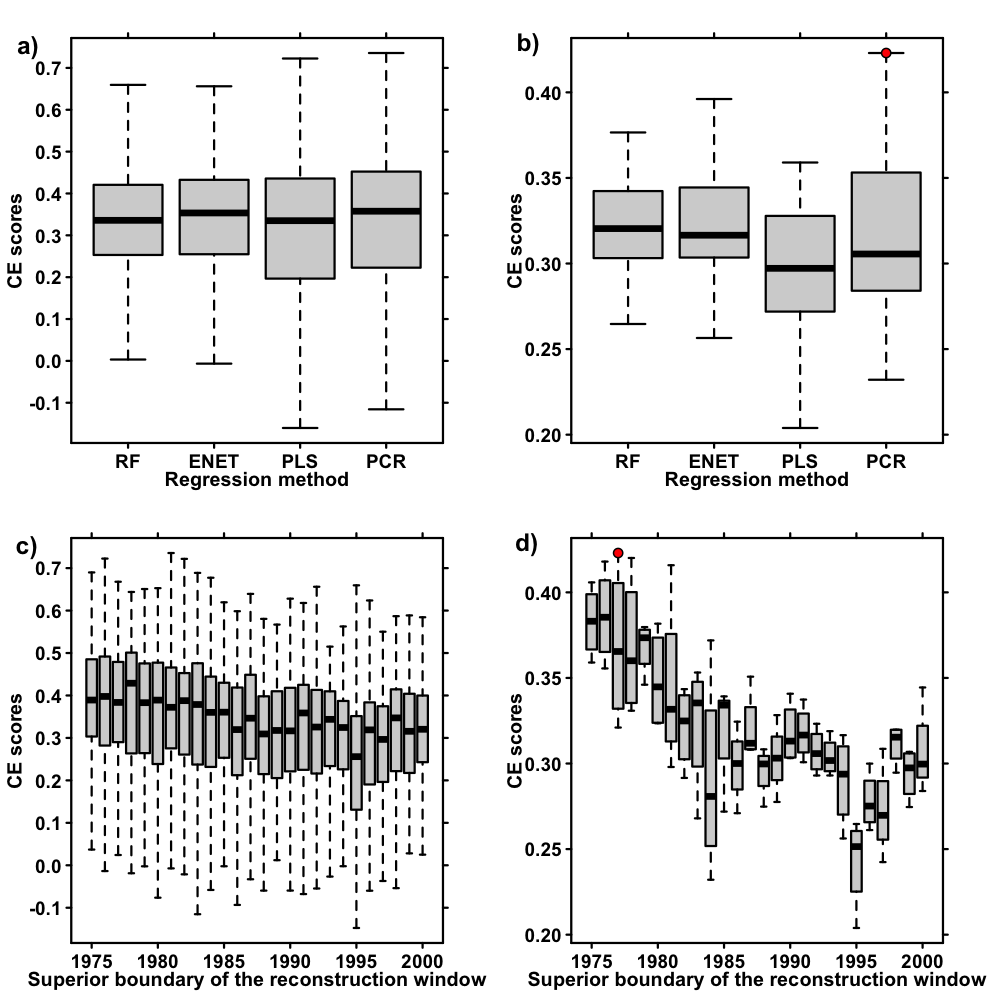


Supplementary Fig. 11. **Score by level of inputs for the 104 North Atlantic Sea Surface Temperature (NASST) reconstructions.** Coefficient of Efficiency scores ($S_{\mathrm{CE}}$) for the 104 reconstructions compared in this study for the different sources of methodological choices (regression method and reconstruction frame). **a** and **b** give the $S_{\mathrm{CE}}$ scores by regression methods (104/4=26 reconstructions by method). **c** and **d** give the $S_{\mathrm{CE}}$ scores by superior boundary of the reconstruction window (104/26=12 final reconstructions by window). **a** and **c** give the $S_{\mathrm{CE}}$ scores for all the training splits (104*30=3,120 scores, respectively 780 and 120 scores by boxplot for the three panels) **b** and **d** give $S_{\mathrm{CE}}$ scores for each final reconstruction as the averages of the 30 corresponding individual $S_{\mathrm{CE}}$ scores (104 average scores). Red dots indicate the highest average score obtained for the particular level of input. For all boxplots, medians are shown as heavy dark lines. Boxplots edges give first and third quartiles. Boxplot “whiskers” give the 10%-90% range. Outliers are not shown. A point from a boxplot is here considered as an outlier when it is outside 1.5 times the interquartile range above the upper quartile and below the lower quartile.

**
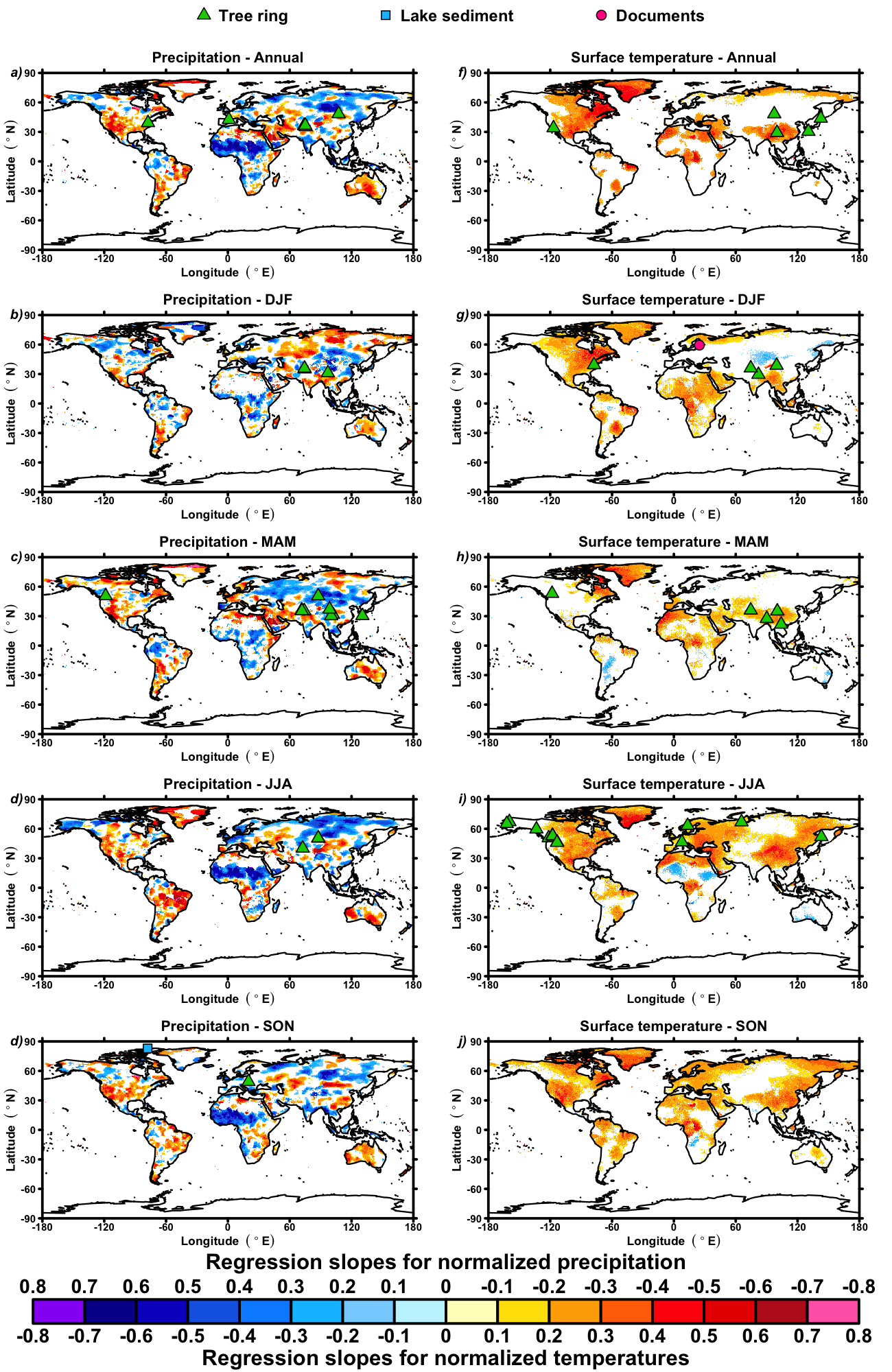
**

Supplementary Fig. 12. **Location of proxy records and Atlantic Multidecadal Variability (AMV) climate fingerprint.** Same regression maps as Fig. 1 but with the location of the 54 (out of 55) terrestrial proxy records used for the reconstruction presented in this study. Tree ring, lake sediment, and document proxies are indicated as green triangles, blue squares, and red circles, respectively.


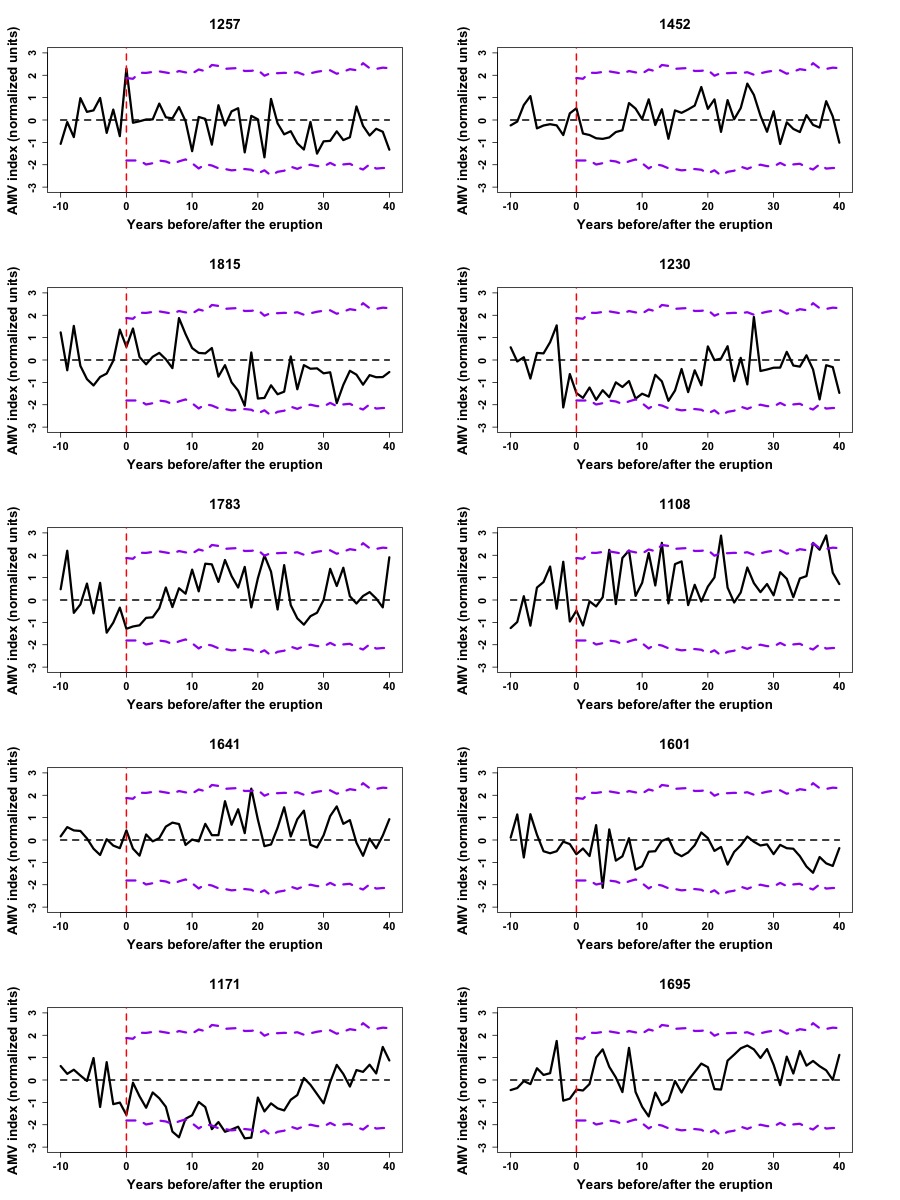


Supplementary Fig. 13. **Individual Atlantic Multidecadal Variability (AMV) responses to the largest volcanic eruptions of the last millennium^38^.** The year of the corresponding eruption is indicated above each panel. For each panel, the sub-time series of the AMV are centered to their average of the 10 years preceding the eruption. Two-tailed 95% confidence levels, calculated using a Monte-Carlo sampling^45^ of length 1000, are given by the dashed purple lines (see Methods).


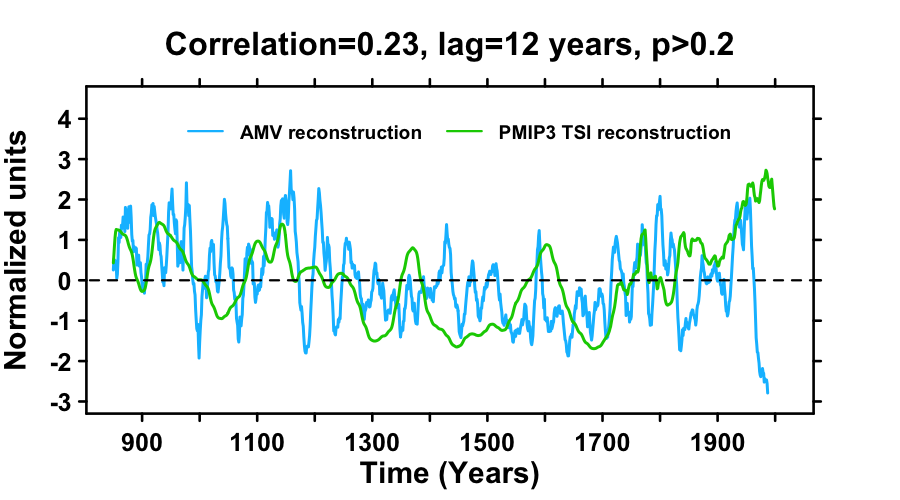


Supplementary Fig. 14. **Comparison of the Atlantic Multidecadal Variability (AMV) reconstruction with the Paleoclimate Modelling Intercomparison Project phase 3 (PMIP3) Total Solar Irradiance (TSI) reconstruction^61^.** Units have been normalized after filtering.


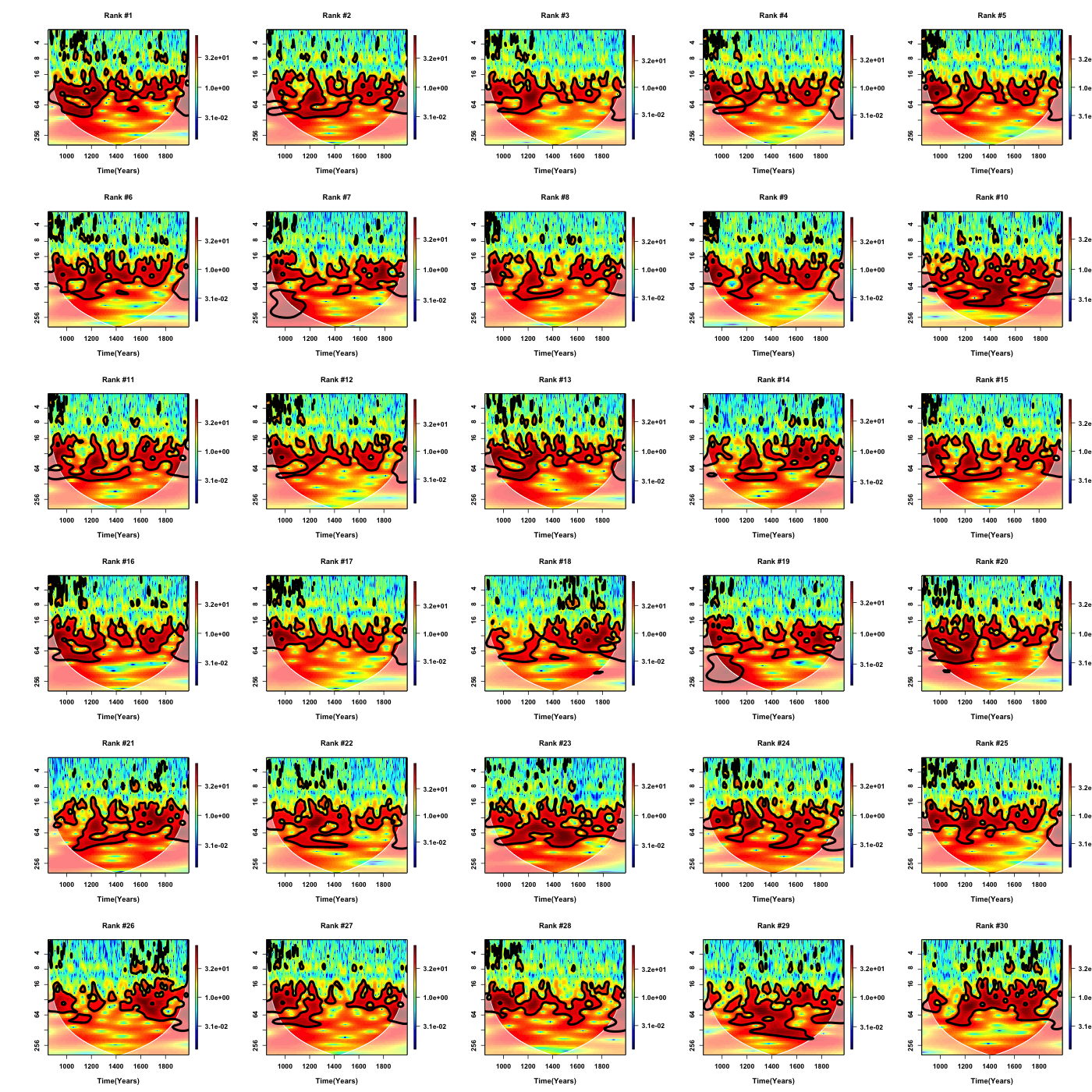


Supplementary Fig. 15. **Composite response of the 30 best Atlantic Multidecadal Variability (AMV) reconstructions.** Same as Fig. 4 but for the 30 best reconstructions (Supplementary Fig. 6)


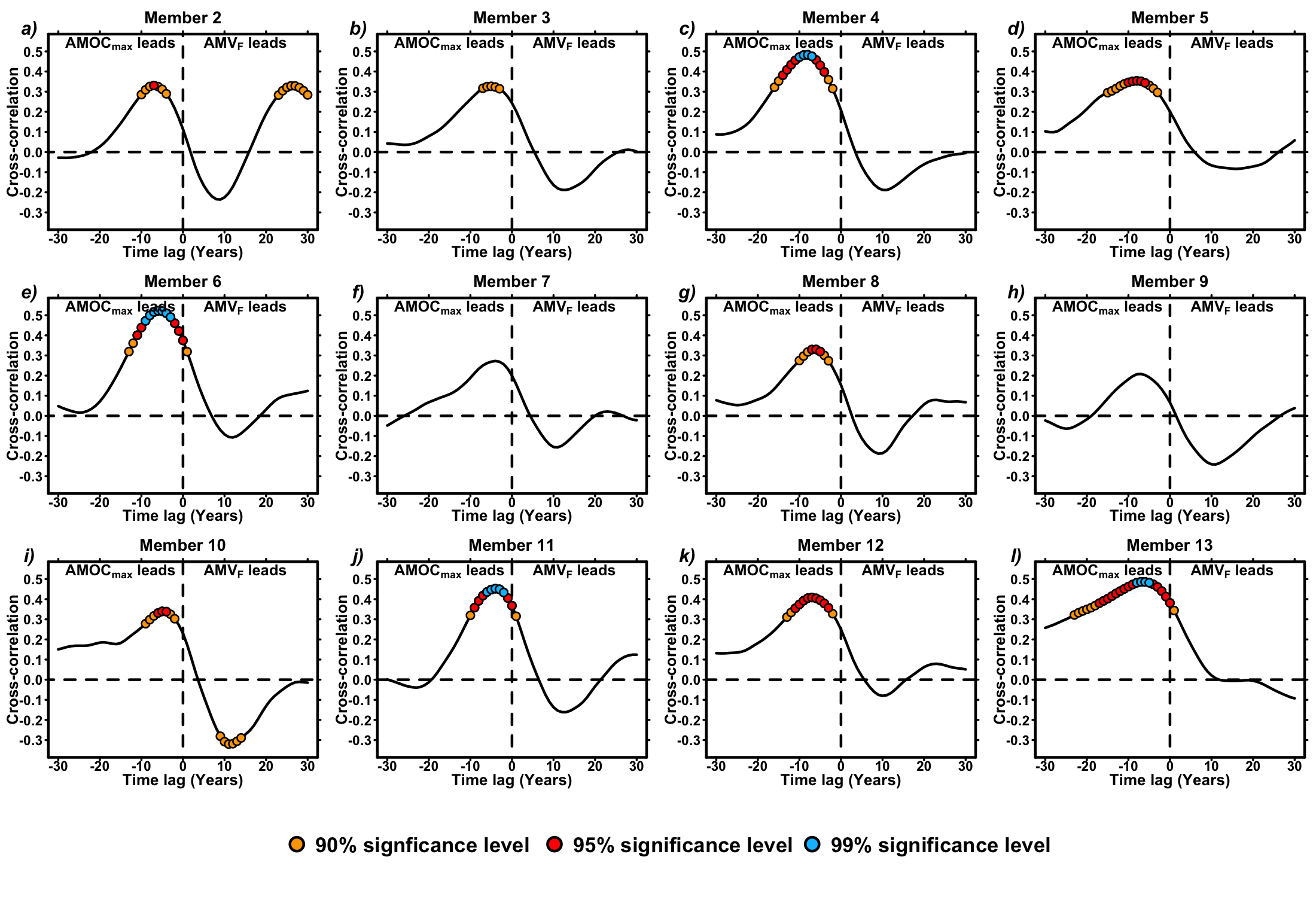


Supplementary Fig. 16. **Cross-correlation functions between the maximum Atlantic Meridional Overturning Circulation strength below 500-meter depth (AMOC_max_) and the the Atlantic Multidecadal Variability index (AMV_F_) in 12 Community Earth System Model Last Millennium Ensemble (CESM-LME) members.** The AMOC_max_ index leads for negative time lags. Significant lagged correlations are indicated with orange dots (90% confidence level), red dots (95% confidence level), and blue dots (99% confidence level).

**
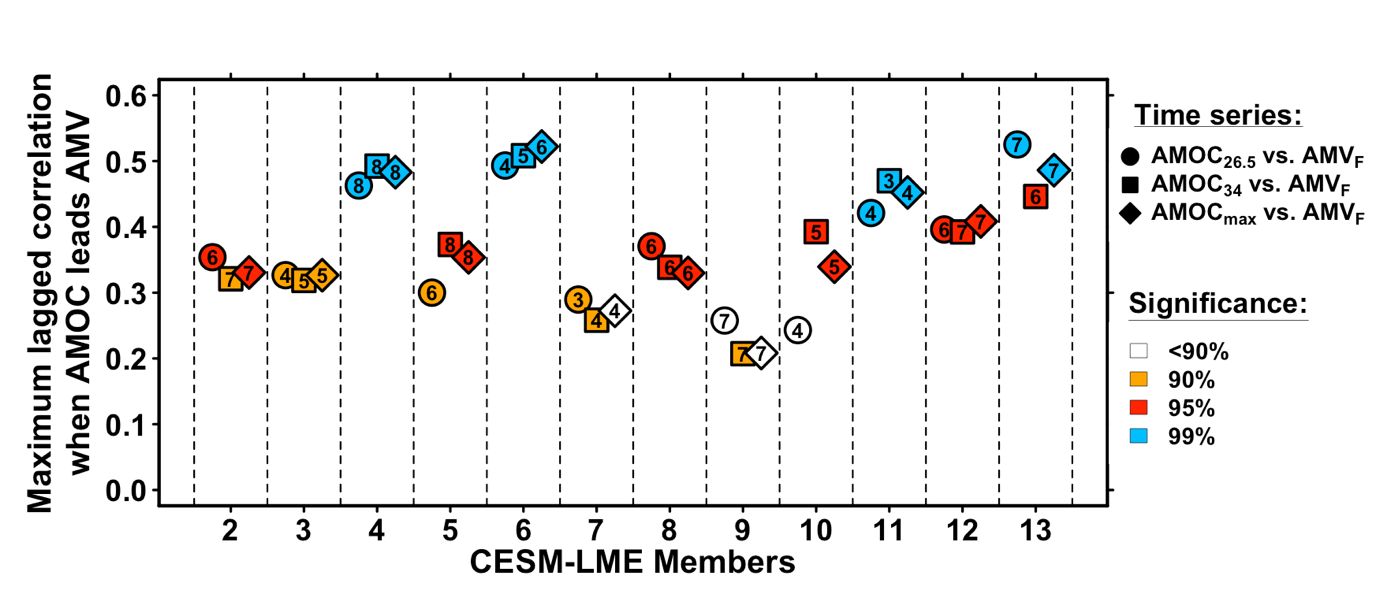
**

Supplementary Fig. 17. **Maximum lagged correlations when 3 Atlantic Meridional Overturning Circulation strength indices lead the AMV_F_ index (AMV_F_) in 12 Community Earth System Model Last Millennium Ensemble (CESM-LME) members**. Circles, squares and diamonds correspond to the maximum lagged correlation when the maximum stream functions below 500 meters at 26°N (AMOC_26_), 34°N (AMOC_34_), and the AMOC_max_ index leads the AMV_F_ index, respectively. Orange, red and blue colors indicate 90%, 95%, and 99% confidence levels, respectively. White points indicate no significance at the 90% confidence level. Numbers in each point indicate the time step where the maximum cross-correlation is reached. All time series are smoothed with a 10-year kernel filter.

**
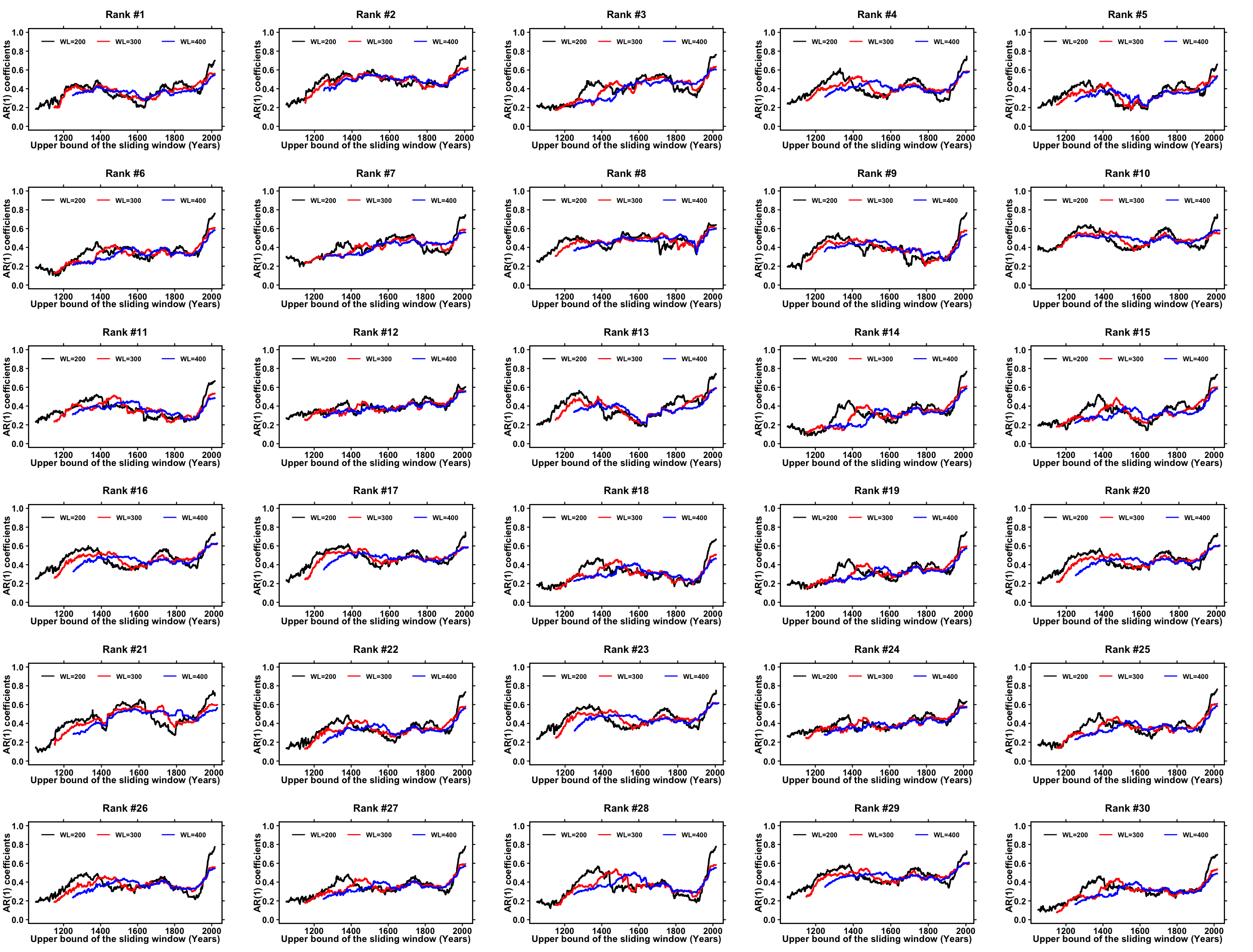
**Supplementary Fig. 18. **Early warning signals test for the 30 best Atlantic Multidecadal Variability (AMV) reconstructions^39,40^.** Same as Fig. 6a but for the 30 best reconstructions (Supplementary Fig. 3). As compared to Fig. 6a, Early warning signals have been computed for three window lengths: 200 (black lines), 300 (red lines) and 400 (blue lines).

**Supplementary Note 1: Regression Methods**

In the general case, we denote $\mathbf{Y}\in\mathbb{R}^{n}$ the predictand, which corresponds to a given AMV index. $\mathbf{x}\in\mathbb{R}^{n\times p}$ corresponds to a given matrix of $p$ proxies observed for the same $n$ time steps than $\mathbf{Y}$, namely a portion of the historical period in our case. We then denote $\mathbf{x}^{\mathbf{'}}\in\mathbb{R}^{m\times p}$, a matrix of $m$ other observations in the past of the same $p$ proxy records for which we search to reconstruct $\mathbf{Y}$. Each regression method has to be optimized for one or more of its so-called control parameters_­_^58^ (or hyperparameters). Control parameters are technical selections inherent to each regression method making a statistical model to act differently when fitting a given set of training data. For example, for PCR, the reconstruction is highly sensitive to the number of principal components used for regressing the climate index^58,62,63^. For each method presented below, each of their control parameters will be identified. However, the exact tuning method used for all regression approaches, namely the k-fold cross-validation, is explained in the Methods section and ref. 58.

Principal Component Regression (PCR):

The PCR^64^ consists in finding the best linear combination to regress $\mathbf{Y}$ using the Principal Components^64^ (PCs) of $\mathbf{x}$. We denote $\mathbf{S}=\mathbf{x}^{T}\mathbf{x}\in\mathbb{R}^{p\times p}$, the variance matrix of $\mathbf{x}$, where $\mathbf{x}^{T}$ is the transposed matrix of $x$. The eigenvectors of S, or Empirical Orthogonal Functions (EOFs), denoted $\mathbf{V}={(\mathbf{v}^{j})}_{1\leq j\leq p} \in\mathbb{R}^{p\times p}$, are obtained by diagonalizing $\mathbf{S}$, which is equivalent to maximizing the variance of the projection of $\mathbf{x}$ on $\mathbf{V}$^64^ vectors (*i.e.*, the EOFs), with recursively adding an orthogonality constraint. In other words, $\mathbf{V}$ vectors can be calculated as:

$$\mathbf{v}^{1}=\arg\max_{\begin{aligned} \mathbf{v}\in\mathbb{R}^{p} \\ \left| \mathbf{v} \right|=1 \end{aligned}} \mathrm{Var}(\mathbf{xv})$$

$$\mathbf{v}^{2}=\arg\max_{\begin{aligned} \mathbf{v}\in\mathbb{R}^{p} \\ \left| \mathbf{v} \right|=1 \\ \mathbf{v}^{T}\mathbf{v}^{1}=0 \end{aligned}} \mathrm{Var}(\mathbf{xv})$$

$$\ldots$$

$$\mathbf{v}^{p}=arg\max_{\begin{aligned} \mathbf{v}\in\mathbb{R}^{p} \\ \left| \mathbf{v} \right|=1 \\ \mathbf{v}^{T}\mathbf{v}^{1}=0 \\ \ldots\\ \mathbf{v}^{T}\mathbf{v}^{p-1}=0 \end{aligned}} Var(\mathbf{xv})$$

Importantly, resolving the above equations is often preferred than directly diagonalizing $\mathbf{S}$. This allows to calculate the PCs even in a case where $p>n$, meaning that $\mathbf{S}$ is not invertible.

The PC matrix denoted $\mathbf{U}={(\mathbf{u}^{j})}_{1\leq j\leq p}$ is calculated by projecting $\mathbf{x}$ on $\mathbf{V}$: $\mathbf{U}=\mathbf{xV}$. Using k-fold cross validation^­58,59^, we determine $q<p$ PCs kept for the regression, which is the control parameter here. The PCR model is then constructed by estimating the best linear regression between $(\mathbf{u}^{1},\ldots,\mathbf{u}^{q})$ and $Y$. The linear regression model is:

$$\mathbf{Y}=\boldsymbol{\beta}_{0}+\sum_{1\leq j\leq q} \boldsymbol{\beta}_{j}\mathbf{u}^{j}+\boldsymbol{\varepsilon}$$

Here, $\boldsymbol{\varepsilon}\in\mathbb{R}^{n}$ is a vector of gaussian white noise with same $\sigma^{2}$ variances. The regression coefficients, $\boldsymbol{\beta}={(\boldsymbol{\beta}_{k})}_{0\leq k\leq q}$, are estimated by $\hat{\boldsymbol{\beta}}={({\hat{\boldsymbol{\beta}}}_{k})}_{0\leq k\leq q}$, given by the ordinary least squares estimator:

$$\hat{\boldsymbol{\beta}}=\arg\min_{\boldsymbol{\beta}\in\mathbb{R}^{q+1}} \mathbf{Y}-\boldsymbol{\beta}_{0}+\sum_{1\leq j\leq q} \boldsymbol{\beta}_{j}\mathbf{u}^{j}=\arg\min_{\boldsymbol{\beta}\in\mathbb{R}^{q+1}} \boldsymbol{\varepsilon}={(\mathbf{u'}^{T}\mathbf{u'})}^{-1}\mathbf{u'Y}$$

Where $\mathbf{u}^{\mathbf{'}}=(\mathbf{1}_{n},\mathbf{u})$, with $\mathbf{1}_{n}$ an all-ones vector of length $n$.

The extended AMV index is obtained by applying the estimated regression coefficients to the projected new observations on the EOFs (*i.e.,* $\mathbf{u}^{\mathbf{'}}=\mathbf{x'V}$):

$$\hat{\mathbf{Y}}={\hat{\boldsymbol{\beta}}}_{0}+\sum_{1\leq j\leq q} {\hat{\boldsymbol{\beta}}}_{j}\boldsymbol{u'}^{j}$$

Partial Least Squares (PLS):

The PLS regression^65^ is a variant approach of the PCR, where the EOFs are calculated such that they are orthogonal and that the covariance between $\mathbf{Y}$ and the projection of $\mathbf{x}$ on the EOFs is maximized. To do so, we need to resolve the following equations:

$$\mathbf{v}^{1}=\arg\max_{\begin{aligned} \mathbf{v}\mathbb{\in R} \\ \left| \mathbf{v} \right|=1 \end{aligned}} \mathrm{Cov}(\mathbf{Y},\mathbf{xv})$$

$$\mathbf{v}^{2}=\arg\max_{\begin{aligned} \mathbf{v}\mathbb{\in R} \\ \left| \mathbf{v} \right|=1 \\ \mathbf{v}^{T}\mathbf{v}^{1}=0 \end{aligned}} \mathrm{Cov}(\mathbf{Y},\mathbf{xv})$$

$$\ldots$$

$$\mathbf{v}^{p}=\arg\max_{\begin{aligned} \mathbf{v}\in\mathbb{R}^{p} \\ \left| \mathbf{v} \right|=1 \\ \mathbf{v}^{T}\mathbf{v}^{1}=0 \\ \ldots\\ \mathbf{v}^{T}\mathbf{v}^{p-1}=0 \end{aligned}} \mathrm{Cov}(\mathbf{Y},\mathbf{xv})$$

Analogously to the PCR, the latent variables here (LVs; PCs analog in PLS) are calculated by projecting $x$ on the matrix: $\mathbf{V}={(\mathbf{v}^{j})}_{1\leq j\leq p} \in\mathbb{R}^{p\times p}, \mathbf{U}=\mathbf{xV}={(\mathbf{u}^{j})}_{1\leq j\leq p}\in\mathbb{R}^{n\times p}$. Using the KFCV optimization (cf. Methods, section “k-fold cross-validation (KFCV)”, we determine $l\leq p$ LVs kept for the regression, which is the only control parameter for PLS. We then construct the regression model by estimating the best linear regression between $\mathbf{u}^{1},\ldots,\mathbf{u}^{l}$ and $\mathbf{Y}$:

$$\mathbf{Y}=\boldsymbol{\beta}_{0}+\sum_{1\leq k\leq l} \boldsymbol{\beta}_{j}\mathbf{u}^{k}+\boldsymbol{\varepsilon}$$

As for the PCR, $\boldsymbol{\beta}={(\boldsymbol{\beta}_{k})}_{0\leq k\leq q}$, are estimated by $\hat{\boldsymbol{\beta}}={({\hat{\boldsymbol{\beta}}}_{k})}_{0\leq k\leq q}$, given by the ordinary least squares estimator:

$$\hat{\boldsymbol{\beta}}=\arg\min_{\boldsymbol{\beta}\in\mathbb{R}^{l+1}} \mathbf{Y}-\boldsymbol{\beta}_{0}+\sum_{1\leq k\leq l} \boldsymbol{\beta}_{j}\mathbf{u}^{k}=\arg\min_{\boldsymbol{\beta}\in\mathbb{R}^{l+1}} \boldsymbol{\varepsilon}={(\mathbf{u'}^{T}\mathbf{u'})}^{-1}\mathbf{u'}Y$$

Where $\mathbf{u}^{\mathbf{'}}=(\mathbf{1}_{n},\mathbf{u})$, with $\mathbf{1}_{n}$ an all-ones vector of length $n$.

The extended AMV index is obtained by applying the estimated regression coefficients to the projected new observations on the EOFs (i.e.*,* $u^{'}=\mathbf{x'V}$):

$$\hat{\mathbf{Y}}={\hat{\boldsymbol{\beta}}}_{0}+\sum_{1\leq k\leq l} {\hat{\boldsymbol{\beta}}}_{j}\mathbf{u'}^{k}$$

Elastic Net regression (Enet):

In the multiple regression case, $\hat{\boldsymbol{\beta}}={({\hat{\boldsymbol{\beta}}}_{k})}_{0\leq k\leq p}$ is estimated by the Ordinary Least Squares estimator. This usual regression coefficient estimator has nevertheless some limitations. Indeed, it is known to often result in a poor reconstruction accuracy due to several assumptions made on the original data, such as homoscedasticity. Thus, previous studies developed regularized regression approaches^66-68^ to overcome the default from the ordinary least squares estimator. The Enet regression^66^ is a combination of the so-called Ridge^67^ and Lasso^68^ regression models. The Ridge regression shrinks towards zero the estimated coefficients associated to predictors unlinked to the predictand. By contrast, Lasso also reduces the variability of the estimates, but in this case by shrinking to zero the estimated coefficients associated with unreliable predictors. Hence, a selection is made by rejecting predictors associated to coefficients shrunk to zero

A regularized, or penalized, regression adds a threshold constraint using the $l_{k}$ norm of $\boldsymbol{\beta}: \left\| \boldsymbol{\beta} \right\|_{k}^{k}=\sum_{1\leq j\leq k} \left| \boldsymbol{\beta}_{j} \right|^{k}$. With $k=1$ for Lasso and $k=2$ for Ridge. The loss function of Elastic Net is given by:

$$L^{\mathrm{enet}}\left( \boldsymbol{\beta} \right)=\left\| \mathbf{Y}-\sum_{1\leq j\leq p} \boldsymbol{\beta}_{j}\mathbf{x}^{j} \right\|_{2}^{2}+\lambda_{1}\sum_{1\leq j\leq p} \left| \boldsymbol{\beta}_{j} \right|+\boldsymbol{\lambda}_{2}\sum_{1\leq j\leq p} \boldsymbol{\beta}_{j}^{2}$$

Where $\lambda_{1}$ and $\lambda_{2}$ are penalty factors.

Let $\mathbf{w}={(\mathbf{w}_{j})}_{1\leq j\leq p}={({sgn(\boldsymbol{\beta}}_{j}))}_{1\leq j\leq p}$ where $sgn(\cdot)$ is the sign function. The loss function of the Enet model can then be denoted as:

$$L^{\mathrm{enet}}\left( \boldsymbol{\beta} \right)=\left\| \mathbf{Y}-\boldsymbol{x\beta} \right\|_{2}^{2}+\lambda_{1}\mathbf{w}^{T}\boldsymbol{\beta}+\lambda_{2}\boldsymbol{\beta}^{T}\boldsymbol{\beta}$$

The estimated coefficients are obtained by minimizing the loss function:

$${\hat{\boldsymbol{\beta}}}^{\mathrm{enet}}={{(\mathbf{x}}^{T}\mathbf{x}+(1-\alpha)\lambda\mathbf{I})}^{-1}{(\mathbf{x}}^{T}\mathbf{Y}-\frac{\alpha\lambda}{2}\mathbf{w})$$

Where $\alpha\in[0,1]$. If $\alpha=1$, a Ridge regression model is applied, while if $\alpha=0$, a Lasso regression model is applied. Otherwise, a combined regression model is applied (*i.e.*, the Elastic Net model). Of note, this choice is very important since it picks the best regression method among the three (*i.e.*, Lasso, Ridge, and Enet). The parameter $\alpha$ thus controls the relative balance between the penalizations associated with $l_{1}$ and $l_{2}$ norms. The parameter $\lambda$ the global intensity of the penalization in the model.

The reconstruction is obtained by applying the estimated regression coefficients ${\hat{\boldsymbol{\beta}}}^{enet}$ on $\mathbf{x}^{1},\ldots,\mathbf{x}^{p}$:

$${\hat{\mathbf{Y}}}_{\lambda,\alpha}=\sum_{1\leq j\leq p} \mathbf{x}^{j}{\hat{\boldsymbol{\beta}}}_{j}^{enet}$$

The optimization of $\alpha$ and $\lambda$ is performed using KFCV (*cf.* Methods, section “k-fold cross-validation (KFCV)”) for both by crossing their possible values. Since they both take their values in continuous sets (*i.e.* $[0,1]$ and $\mathbb{R}^{p}$), they have to be discretized. The more they are, the more robust the reconstruction will be, at the expanse of the computational time.

Random Forest (RF):

The RF regression has been introduced in the early 21^st^ century^69^ and consists in aggregating regression trees, which will be defined first.

We denote the $\{{{(\mathbf{Y}}_{i},\mathbf{x}_{i})}_{1\leq i\leq n}\}$ each observation of $\mathbf{Y}$ and $\mathbf{x}$ for each time step. The first step of the regression tree algorithm consists in finding among the $p$ records, which is the best in cutting $\mathbf{Y}$ into a partition of two groups, such that the sum of the intra-group variances is minimal. Those groups are called the child nodes (*i.e.*, left and right) of the root $\left\{ {{(\mathbf{Y}}_{i},\mathbf{x}_{i})}_{1\leq i\leq n} \right\}$. To draw these groups, we call any cut of the data by:

$$\left\{ i:\mathbf{x}_{i}^{j}<d \right\}\cup\left\{ {i:\mathbf{x}}_{i}^{j}>d \right\}$$

Where $d$ is the threshold values such that any year of observation of the $j^{\mathrm{th}}$ variable which is higher than $d$ is put into the right child node, whereas years of observation lower than $d$ is put into the left child node. Finding the optimal cut of $\left\{ {{(\mathbf{Y}}_{i},\mathbf{x}_{i})}_{1\leq i\leq n} \right\}$ thus consists of resolving the following convex optimization problem:

$$\left( j,d \right)=\arg\min_{\begin{aligned} d\mathbb{\in R} \\ 1\leq j\leq p \end{aligned}} \sum_{i:\mathbf{x}_{i}^{j}<d} {(\mathbf{x}_{i}-\frac{1}{\#(\left\{ i:\mathbf{x}_{i}^{j}<d \right\})})\sum_{i:\mathbf{x}_{i}^{j}<d} \mathbf{Y}_{i})}^{2}+\sum_{i:\mathbf{x}_{i}^{j}>d} {(\mathbf{Y}_{i}-\frac{1}{\#(\left\{ i:\mathbf{x}_{i}^{j}>d \right\})})\sum_{i:\mathbf{x}_{i}^{j}>d} \mathbf{Y}_{i})}^{2}$$

Afterwards, the same algorithm is applied to the child nodes, and so on recursively. Once the three is built each node without child node is called a leaf. A leaf can be a single value of $\mathbf{Y}$ (if only one observation remains), or a realization of the distribution of $\mathbf{Y}$ within this leaf^70^. Of note, building regression trees to their maximal depth (*i.e.*, one observation by leaf) is equivalent to building a $k$-nearest-neighbors (or analog) model with $k=1$, which is a very basic statistical model with poor performance^52^. To make regression trees to distinguish from the $1$-nearest-neighbor method, it stops criterion needs to be prescribed. We thus stop the regression tree once one node contains a number of $s$ (or less) years of observations^70^.

The major limitation of regression trees is that they overfits strongly the data with which they are built, often resulting in quite poor reconstruction (or prediction) ability. Hence, considering a learning sample $\{{{(\mathbf{Y}}_{i},\mathbf{x}_{i})}_{1\leq i\leq n}\}$, adding or removing a few variables (*i.e.*, paleoclimate records, columns of $\mathbf{x}$) can strongly modify a given tree and thus the reconstruction (or prediction) it gives. Indeed, if we just remove the first variable selected to cut the root, the resulting tree would be a very different one^69^. In other words, although a regression tree would be an efficient model for some cases, it is too dependent on the data it is built with to be considered as a reliable and robust regression approach nowadays^69^. It is from this observation that random forest was proposed^69^. The idea is to generate a large number of regression trees (hence the term “forest”), all based on a randomly drawn sample of $m$ of the $p$ intial variables with $m<p$ (hence the term “random”).

Once $B$ trees have been built, the reconstruction (or prediction) of $\mathbf{Y}$ for a given by using values of $\mathbf{x'}$ to browse each of the trees, previously built using valued of $\mathbf{x}$. For a given tree denoted $b$, where $\mathbf{x'}_{i}$ ends up within the leaf $\mathcal{L}$ for year $i$ in the past, the reconstruction is given by the average of $\mathbf{Y}$ within $\mathcal{L}$:

$${\hat{\mathbf{Y}}}_{i}^{(b)}=\frac{1}{\mathcal{\#\{L\}}}\sum_{k:\{\mathbf{Y}_{k},\mathbf{x}_{k}\mathcal{\}\in L}} \mathbf{Y}_{k}$$

The final RF reconstruction for year $i$ is finally given by averaging reconstructions given by the trees, denoted ${\hat{\mathbf{Y}}}_{i}^{(1)},\ldots,{\hat{\mathbf{Y}}}_{i}^{(B)}$:

$${\hat{\mathbf{Y}}}_{i}=\frac{1}{B}\sum_{1\leq b\leq B} {\hat{\mathbf{Y}}}_{i}^{(b)}$$

By the only fact of generating a large number of trees, the method can be quite costly in terms of computing time and energy use. This is particularly true since trois parameters can be identified ($B,s$ and $m$), making the optimization via cross validation extremely costly. Fortunately, ref. 69 showed that only the optimization of $m$ (*i.e.*, the number of randomly drawn variables for each tree) is worth it. Ref. 69 and 71 thus showed that setting $s=5$ and $B=128$ leads converging RF reconstructions for most datasets, while their optimization would multiply thecomputation time by hundreds of time. Thus only $m$ needs to be optimized by k-fold cross validation (*cf.* Methods, section “k-fold cross-validation (KFCV)”) for the RF method.

**Supplementary Note 2: Comparison with independent ocean proxy records**

We note that as in previous studies reconstructing the AMV^62,63^, most of the proxy records used here are terrestrial, which might be surprising given that the AMV is an oceanic mode. Our objective method only includes a single oceanic P2k+ proxy from the 55 selected, mainly because ocean proxies typically have a resolution that is lower than annual, our first requisite for proxy records pre-screening (see Methods). To assess whether our AMV reconstruction is in agreement with existing ocean proxy records, we test its correlation with independent coral and sediment-based ocean proxy records from the Ocean 2k database^36^ which were not used in the reconstruction. Because some of these proxy records have very low temporal resolutions, which could lead to spurious significant correlations with the AMV, we use a significance test that takes into account time series autocorrelations^62,63^ (see Methods). It appears that 37 ocean records (23 from the North Atlantic, including the Mediterranean Sea) from the Ocean 2k^36^ database are significantly correlated with our AMV reconstruction, at least at the 90% confidence level (Supplementary Fig. 8a). For further validation, we compute two composite time series of these selected ocean proxy data (see Methods). The first is an aggregate based on the average of the 37 ocean proxies  (Supplementary Fig. 8a), and the second is based solely on the 23 North Atlantic proxies from (Supplementary Fig. 8a,b). We find strong and significant correlations between the 30-year filtered AMV reconstruction and these composite time series based on all ocean proxies from Supplementary Fig. 8a (r=0.75, p<0.01) and also with only those from the North Atlantic (r=0.76 p<0.01, Supplementary Fig. 8b), thus further supporting the robustness of our nested reconstruction (Supplementary Fig. 8a,b).

**Supplementary Note 3: Pseudo-proxy experiments**

These supplementary notes detail the outputs of the pseudo-proxy experiments carried out for this study. Since this part is quite technical and need space to properly study the outputs, this part from the Supplementary remain a very important validation for our reconstruction which highly strengthen its reliability and robustness. Although results from PPE are fully detailed here, some technical aspects are also given in the Methods section of the main manuscript.

A PPE consists in using climate model simulations to evaluate if the approach used here can allow reconstructing the AMV from the model outputs, taking as “pseudo-proxies” the modelled climate variables at the spatial location of each real-world proxy (Methods). For this purpose, we use 12 members from the Last Millennium Large Ensemble of the Community Earth System Model 1 (CESM-LME, see Methods). Here, we conduct two types of PPE (Methods).

The first PPE consists in exactly reproducing the previous reconstruction from the real-world in the model simulations. For this, we train statistical models over the same period as above (1870-1987), with the time series of the nearest corresponding grid points to the real-world proxy records for each timeframe of the nested reconstruction. The validation then consists in comparing the reconstructed last millennium AMV_F_ in the model (hereafter PPE-reconstructed-AMV) to the AMV_F_ effectively simulated by the model (hereafter, the model-AMV). For each member of CESM1-LME, we calculated the correlation between the model-AMV and the PPE-reconstructed-AMV (Supplementary Fig. 9). All of them are significant at the 90% confidence level for the 10-year smoothed time series (r ϵ [0.41,0.57] for the 12 members). In terms of skill scores we find that the real-world median skill score (med($S_{\mathrm{CE}}$)=0.29) falls within the range of those from the PPE, which are significantly positive at the 95% confidence for all the members except for member number 7 (med($S_{\mathrm{CE}}$)=[0,0.6], Supplementary Fig. 9a). This first step of PPE validation therefore provides further confidence in the capabilities of our methodology and constitutes the first model-based validation for an AMV reconstruction.

The second experiment consists in adding constraints from the model on the AMV reconstruction based on real-world proxy records. It consists in first sub-selecting pseudo-proxies according to the correlation between the simulated AMV and the 55 pseudo-proxies from the previous PPE over the whole millennium, allowing us to evaluate stationarity in the linkages in a long-enough timeframe. Here, we further train the RF models within the climate model simulations over the longest period covered by the real-world proxy records, and then apply these RF models to those same records (see Methods). The resulting AMV reconstructions are thus based on AMV/pseudo-proxies relationships from the model simulation over a longer timeframe than the instrumental period. Thus, this approach improves the robustness of the statistical relationships in time, with a better account of the potential non-stationarity properties of the AMV/proxies covariations. These model-constrained reconstructions based on single members are compared to the former AMV_F_ reconstruction based on observations only. They show strong correlations, significant at least at the 95% level for each of the 12 members (r ϵ [0.53,0.88], Supplementary Fig. 10a). The $S_{\mathrm{CE}}$ skill scores are also significantly positive for this PPE (med($S_{\mathrm{CE}}$) ϵ [0.2,0.35], p<0.01 for the 12 members, Supplementary Fig. 10a), which indicates a satisfactory level of robustness for the RF models trained in the CESM1-LME members. The correlation of the ensemble mean of model-constrained AMV reconstructions with the observation-constrained AMV reconstruction is also highly significant (r=0.88, p<0.01). This second step of PPE therefore indicates that training RF models within the millennial-long simulations of the CESM1-LME reproduce very similar reconstructions as the observation-constrained one, highlighting a limited role of potential non-stationarity between AMV signal and proxy records used.

A final key element that supports the validity of the reconstruction, is the fact that the network of proxy records used in the reconstruction has similar weights in both PPE experiments: large weights are not restricted to North Atlantic bordering regions, they also occur in central to eastern Asia and western North America (Supplementary Fig. 9,10), in agreement with the teleconnections highlighted in numerous studies^72-76^.

**References:**

1. Andersen, K., Ditlevsen, P., Rasmussen, S., Clausen, H., Vinther, B., Johnsen, S. & Steffensen, J. Retrieving a common accumulation record for the past 1,800 years. *Journ. Geophys. Res.* **111**, D15106 (2006).
2. Cuffey, K. M., Clown, G. D., Alley, R. B., Stuiver, M., Waddington, E. D. & Saltus, R. W. 6 Large Arctic temperature change at the Wisconsin-Holocene glacial transition. *Science* **270**, 455-458 (1995).
3. Fisher, D. A., Koerner, R. M. & Reeh, N. Holocene climatic records from Agassiz Ice Cap, Ellesmere Island, NWT, Canada. *The Holocene* **33**, 19-24 (2010).
4. Meeker, L. D. & Mayewski, P. A. A 1400-year high-resolution record of atmospheric circulation over the North Atlantic and Asia. *The Holocene* **12**, 257-266 (2002).
5. Büntgen, U., Frank, D. C., Nievergelt, D & Esper, J. Summer temperature variations in the European Alps, A.D. 755-2004. *J. Clim.* **19(21)**, 5606-5623 (2006).
6. Stahle, D. W. International tree-ring Data Bank. AR050. *https://www.ncdc.noaa.gov/data-access/paleoclimatology-data/datasets/tree-ring* (1996).
7. Stahle, D. W. & Cleaveland, M. K. International tree-ring Data Bank. AR052. *https://www.ncdc.noaa.gov/data-access/paleoclimatology-data/datasets/tree-ring* (2005).
8. Tosh, R. International tree-ring Data Bank. CA051. *https://www.ncdc.noaa.gov/data-access/paleoclimatology-data/datasets/tree-ring* (1994).
9. Bunn, A. G., Graumlich, L. J. & Urban, D. L. Trends in twentieth-century tree growth at high elevations in the Sierra Nevada and White Mountains, USA. *The Holocene* **15**, 481-488 (2005).
10. Woodhouse, C. A. and Brown, P. M. International tree-ring Data Bank. CO572. *https://www.ncdc.noaa.gov/data-access/paleoclimatology-data/datasets/tree-ring* (2006).
11. Stahle, D. W. & Cleaveland, M. K. International tree-ring Data Bank. FL001. *https://www.ncdc.noaa.gov/data-access/paleoclimatology-data/datasets/tree-ring* (2005).
12. Stahle, D. W. International tree-ring Data Bank. LA001. 32 *https://www.ncdc.noaa.gov/data-access/paleoclimatology-data/datasets/tree-ring* (1996).
13. Esper, J., Frank, D., Büntgen, U., Verstege, A., Luterbacher, J. & Xoplaki, E. Long-term drought severity variations in Morocco. *Geophys. Res. Lett.* **34**, L17702 (2007).
14. Graumlich, L. J., Pisaric, M. F. J., Waggoner, L. A., Littell, J. S & King, J. C. Upper Yellowstone river flow and teleconnections with Pacific basin climate variability during the past three centuries. *Climatic change* **59**, 245-262 (2003).
15. Touchan, R., Woodhouse, C. A., Meko, D. M. & Allen, C. Millennial precipitation reconstruction for the Jemez Mountains, New Mexico, reveals changing drought signal. Intern. *Journ. Clim.* **31**, 896-906 (2011).
16. Graybill, D. A. International tree-ring Data Bank. NV516. *<https://www.ncdc.noaa.gov/data-access/paleoclimatology-data/datasets/tree-ring>* (1994).
17. Graybill, D. A. International tree-ring Data Bank. NV517. *https://www.ncdc.noaa.gov/data-access/paleoclimatology-data/datasets/tree-ring* (1994).
18. Salzer, M. W. & Kipfmueller, K. F. Reconstructed temperature and precipitation on a millennial timescale from tree-rings in the Southern Colorado plateau, USA. *Climatic chang*e **70**, 465-487 (2005).
19. Naurzbaev, M. M., Vaganov, E. A., Sidorova, O. V. & Schweingruber, F. H. Summer temperatures in eastern Taimyr inferred from a 2427-year late-Holocene tree-ring chronology and earlier floating series. *The Holocene* **12**, 727-736 (2002).
20. Graybill, D. A. International tree-ring Data Bank. UT508. *https://www.ncdc.noaa.gov/data-access/paleoclimatology-data/datasets/tree-ring* (1994).
21. Graybill, D. A. International tree-ring Data Bank. UT509. *https://www.ncdc.noaa.gov/data-access/paleoclimatology-data/datasets/tree-ring* (1994).
22. Stahle, D. K., Brunette, D. J. & Stahle, D. W. A moisture balance reconstruction for the drainage basin of Albermale Sound, North Carolina. *Estuaries and Coasts.* **36**, 1340-1353 (2013).
23. Björklund, J. A., Gunnarson, B. E, Seftigen, K., Esper, J. & Linderholm, H. W. Blue intensity and density from northern Fennoscandian tree rings, exploring the potential to improve summer temperature reconstructions with earlywood information. *Clim. Past.* **10**, 877-885 (2014).
24. Zhang, P., Linderholm, H. W., Gunnarson, B. E., Björklund, J. A. & Chen, D. 1200 years of warm-season temperature variability in central Scandinavia inferred from tree-ring density. *Clim. Past.* **12**, 1297-1312 (2016).
25. McCarroll, D., Loader, N. J., Jalkanen, R. Gagen, M. H., Hakan Grudd, H. & Gunnarson, B. E. Fennoscandia 1200 year tree growth data and summer temperature reconstruction. *The Holocene* **23**, 471-484 (2013).
26. Georges, S. S. & Nielsen, E. Hydroclimatic change in southern Manitoba since A. D. 1409 inferred from tree rings. *Quat. Res.* **58**, 103-111 (2002).
27. Stahle, D. W., Villanueva Diaz, J., Brunette, D. J., Cerano Paredes, J., Heim Jr., R. R., Fye, F. K., Acuna Soto, R., Therell, M. D., Cleaveland, M. K. & Stahle, D. K. Major Mesoamerican droughts of the past millennium. *Geophys. Res. Lett.* **38**, L05703 78 (2011).
28. Andersen, K. K., Bigler, M., Buchardt, S. L., Clausen, H. B., Dahl-Jensen, D., Davies, S. M., Fischer, H., Goto-Azuma, K., Hansson, M. E., Heinemeier, J., Johnsen, S. J., Larsen, L. B., Mischeler, R., Olsen, G. J., Rasmussen, S. O., Röthlisberger, R., Ruth, U., Seierstad, I. K., Siggaard-Andersen, M.-L., Steense, J. P., Svensson, A. M. & Vinther, B. M. Greenland Ice core chronology 2005 (GICC05) and 20 year means of oxygen isotope data from ice core NGRIP. *https://doi.org/10.1594/PANGAEA.586838* (2007).
29. Maxwell, R. S., Hessl, A. E., Cook, E. R. & Pederson, N. A multispecies tree ring reconstruction of Potomac river streamflow (950-2001). *Water Resources Research* **47**, W05512 (2011).
30. Wilson, R., Miles, D., Loader, N. J., Cooper, R. & Briffa, K. A millennial long March-July precipitation reconstruction for southern-central England. *Clim. Dyn.* **40(3-4)**, 997-1017 (2013).
31. Lindholm, M. & Jalkanen, R. Subcentury scale variability in height-increment and tree ring width chronologies of Scots pine since A. D. 745 in northern Finland. *The Holocene* **22**, 571-577 (2011).
32. Helama, S., Holopainen, J., Timonen, M. & Mielikäinen, K. An 854-year tree-ring chronology of Scots Pine for South-West Finland. *Studia Quaternaria.* **31**, 61-68 96 (2014).
33. Touchan, R., Garfin, G. M., Meko, D. M., Funkhouser, G., Erkan, N., Hugues, M. K. & Wallin, B. S. Preliminary reconstructions of spring precipitation in southwestern Turkey from tree ring width. *Int. Journ. Clim.* **23**, 157-171 (2003).
34. Esper, J., Büntgen, U., Frank, D., Verstege, A., Nievergelt, D. & Liebhold, A. 1200 years of regular outbreaks is alpine insects. *Proc. Biol. Sci.* **274(1610)**, 671-679 (2006).
35. Reynolds, D. J., Scourse, J. D., Halloran, P. R., Nederbragt, A. J., Wanamaker, A. D., Butler, P. G., Richardson, C. A., Heinemeier, J., Eiriksson, J., Knudsen, K. L. & Hall, I. R. Annually-resolved North Atlantic marine climate over the last millennium, *Nat. Com.* **7**, 185-189 (2016).
36. PAGES 2k Consortium. A global multiproxy database for temperature reconstructions of the Common Era. *Sci. Data.* **4**, 170088 (2017).
37. Harris, I., Osborn, T. J., Jones, P. & Lister, D. Version 4 of the CRU TS monthly high-resolution gridded multivariate climate dataset. *Sci. Data.* **7(1)**, 109 (2020).
38. Sigl, M., Winstrup, M., McConnell, J. R., Welten, K. C., Plunkett, G., Ludlow, F., Büntgen, U., Caffee, M., Chellman, N., Dahl-Jensen, D., Fisher, H., Kipfstuhl, S., Kostick, C., Maselli, J., Mekhaldi, F., Mulvaney, R., Muscheler, R., Pasteris, D. R., Pilcher, J. R., Salzer, M., Schüpbach, S., Steffensen, J. P., Vinther, B. M. & Woodruff, T. E. Timing and climate forcing of volcanic eruptions for the past 2,500 years. *Nature* **523**, 543-549 (2015)
39. Boulton, C. A., Allison, L. C. & Lenton, T. M. Early warning signals of Atlantic Meridional Overturning Circulation collapse in a fully coupled climate model. *Nature Commun.* **5**, 5752 (2014).
40. Lenton, T. M. Early warning of climate tipping point. *Nature Clim. Change* **1**, 201-208 (2011).
41. Gouhier, T. C., Grinsted, A. & Simko, V. R package biwavelet: Conduct Univariate and Bivariate Wavelet Analyses (Version 0.20.19). Available from <https://github.com/tgouhier/biwavelet> (2019).
42. Simon, N., Friedman, J., Hastie, T. & Tibshirani, R. Regularization Paths for Cox's Proportional Hazards Model via Coordinate Descent. *Journal of Statistical Software* **39(5)**, 1-13. <https://www.jstatsoft.org/v39/i05/> (2011).
43. Warnes, G. R., Bolker, B., Bonebakker, L., Gentleman, R., Huber, W., Liaw, A., Lumley, T., Maechler, M., Magnusson, A., Moeller, S., Schwartz, M. & Venables, B. gplots: Various R Programming Tools for Plotting Data. R package version 3.1.1. <https://CRAN.R-project.org/package=gplots> (2020).
44. Meschiari, S. latex2exp: Use LaTeX Expressions in Plots. R package version 0.4.0. <https://CRAN.R-project.org/package=latex2exp> (2015).
45. Original S code by Becker, R. A. & Wilks, A. R. R version by Brownrigg, R. Enhancements by Minka, T. P. & Deckmyn, A. maps: Draw Geographical Maps. R package version 3.3.0. <https://CRAN.R-project.org/package=maps> (2018).
46. Pierce, D. ncdf4: Interface to Unidata netCDF (Version 4 or Earlier) Format Data Files. R package version 1.17. <https://CRAN.R-project.org/package=ncdf4> (2019).
47. Mevik, B.-H., Wehrens, R. & Liland, K. H. pls: Partial Least Squares and Principal Component Regression. R package version 2.7-3. <https://CRAN.R-project.org/package=pls> (2020).
48. Borchers, H. W. pracma: Practical Numerical Math Functions. R package version 2.2.9. <https://CRAN.R-project.org/package=pracma> (2019).
49. Liaw, A. & Wiener, M. Classification and Regression by randomForest. *R News* **2(3)**, 18-22 (2002).
50. Neuwirth, E. RColorBrewer: ColorBrewer Palettes. R package version 1.1-2. <https://CRAN.R-project.org/package=RColorBrewer> (2014).
51. Bivand, R. S., Pebesma, E. & Gomez-Rubio, V. Applied spatial data analysis with R, Second edition. *Springer, NY.* <https://asdar-book.org/> (2013).
52. Pebesma, E. J. & Bivand, R. S. Classes and methods for spatial data in R. *R News* **5(2)**, <https://cran.r-project.org/doc/Rnews> (2005).
53. Wickham, H. stringr: Simple, Consistent Wrappers for Common String Operations. R package version 1.4.0. <https://CRAN.R-project.org/package=stringr> (2019).
54. Yee, T. W. Vector Generalized Linear and Additive Models: With an Implementation in R. *New York, USA: Springer* (2015).
55. Yee , T. W. & Wild, C. J. Vector Generalized Additive Models. *Journal of Royal Statistical Society, Series B* **58(3)**, 481-493 (1996).
56. Zeileis, A. & Grothendieck, G. zoo: S3 Infrastructure for Regular and Irregular Time Series. *Journal of Statistical Software* **14(6)**, 1-27 (2005).
57. Ting, M., Kushnir, Y., Saeger, R. & Cuihua, L. Forced and internal twentieth-century SST trends in the North Atlantic. *J. Clim.* **22**, 1469-1481 (2009).
58. Michel, S., Swingedouw, D., Ortega, P., Khodri, M., Mignot, J. & Chavent, M. Reconstructing climatic modes of variability from proxy records using ClimIndRec version 1.0. *Geosci. Mod. Dev.* **13**, 841-858 (2020).
59. Nash, J. E. & Sutcliffe, J. V. River flow forecasting through conceptual models part I: A discussion of principles. *J. Clim.* **10**, 282-290 (1970).
60. Wahl, E. R. & Smerdson, J. E. Comparative performance of paleoclimate field and index reconstructions derived from climate proxies and noise-only predictors. *Geophys. Res. Lett.* **39(6)**, L06703 (2012).
61. Vieira, L. E. A., Solanki, S. K., Krikova, N. A. & Usoskin, I. Evolution of the solar irradiance during the Holocene. *Astronom., Astrophys.* **531**, A6 (2011).
62. Gray, S., Graumlich, L., Betancourt, J. & Pederson, G. D. A tree-ring based reconstruction of the Atlantic Multidecadal Oscillation since 1567 A.D. *Geophys. Res. Lett.* **31**, L12205 (2004).
63. Wang, J., Yang, B., Charpentier Ljungqvist, F., Luterbacher, J., Osborn, T. J., Briffa, K. R. & Zorita, E. Internal and external forcing of multidecadal Atlantic variability over the past 1,200 years. *Nature Geosci.* **10**, 512-518 (2017).
64. Jolliffe, I. T. A note on the use of Principal Components in Regression. *Journ. Roy. Stat. Soc C*. **31(3)**, 300-303 (1982).
65. Wold, S., Ruhe, A., Wolf, H., and Dunn III, W. J. The collinearity problem in linear regression. The Partial Least Squares (PLS) approach to generalized inverses. *J. Sci. Stat. Comput.* **5**, 735-743 (1984).
66. Zou, H. and Hastie, T. Regularization and variable selection via the elastic net. *Journ. Roy. Stat. Soc.* **67**, 301-320 (2005).
67. Hoerl, A. E. and Kennard, R. W. Ridge regression: biased estimation of nonorthogonal problems. *Technometrics*. **12**, 55-67 (1970).
68. Tibshirani, R. Regression shrinkage and selection via Lasso. *Journ. Roy. Stat. Soc.* **58**, 267-288 (1996).
69. Breiman, L. Random Forests. *Mach. Learn*. 45, 5-32 (2001).
70. Breiman, L., Friedman, J. H., Olshen, R. and Stone, C. J. Classification and Regression Trees. *Montery, CA: Wadsworth and Brooks* (1984).
71. Oshiro, T. M., Perez, P. S. and Baranauskas, J. A. How many trees in a random forest? *Lect. Note Comp. Sci.* **7376**, 154-168 (2012).
72. Trenberth, K. & Shea, D. Atlantic Hurricanes and natural variability in 2005. *Geophys. Res. Lett.* **33**, L12704 (2006).
73. Ruprich-Robert, Y., Msadek, R., Castruccio, F., Yeager, S., Delworth, T. & Danabasoglu, G. Assessing the climate impacts of the Observed Atlantic Multidecadal Variability Using the GFDL CM2.1 and NCAR CESM1 Global Coupled Models. *J. Clim.* **30(8)**, 2785-2810 (2017).
74. Shi, C., Sun, C., Wu, G., Wu, X., Chen, D., Masson-Delmotte, V., Li, J., Xue, J., Li, Z., Ji, D., Zhang, J., Fan, Z., Shen, M., Shu, L. & Ciais, P. Summer temperature over the Tibetan Plateau modulated by Atlantic Multidecadal Variability. *J. Clim.* **32(13)**, 4055-4067 (2019).
75. Li, J., Li, F., He, S., Wang, H. & Orsolini, Y. J. The Atlantic Multidecadal Variability phase dependence of teleconnection between the North Atlantic Oscillation in February and the Tibetan Plateau in March. *J. Clim.* **34(11)**, 4227-4242 (2021).
76. Monerie, P.-A., Robson, J., Dong, B. & Hodson, D. Role of the Atlantic Multidecadal Variability in modulating East Asian Climate. *Clim. Dynam.* **56**, 381-398 (2021).
